# Spatial subcellular organelle networks in single cells

**DOI:** 10.1038/s41598-023-32474-y

**Published:** 2023-04-01

**Authors:** Mythreye Venkatesan, Nicholas Zhang, Benoit Marteau, Yukina Yajima, Nerea Ortiz De Zarate Garcia, Zhou Fang, Thomas Hu, Shuangyi Cai, Adam Ford, Harrison Olszewski, Andrew Borst, Ahmet F. Coskun

**Affiliations:** 1 Wallace H. Coulter Department of Biomedical Engineering, Georgia Institute of Technology and Emory University, Atlanta, GA USA; 2 Interdisciplinary Bioengineering Graduate Program, Georgia Institute of Technology, Atlanta, GA USA; 3 School of Electrical and Computer Engineering, Georgia Institute of Technology, Atlanta, GA USA; 4 Departamento de Bioingenieria e Ingenieria Aeroespacial, Universidad Carlos III de Madrid, Getafe, Spain; 5 Parker H. Petit Institute for Bioengineering and Bioscience, Georgia Institute of Technology, Atlanta, GA USA

**Keywords:** Organelles, Single-cell imaging, Image processing

## Abstract

Organelles play important roles in human health and disease, such as maintaining homeostasis, regulating growth and aging, and generating energy. Organelle diversity in cells not only exists between cell types but also between individual cells. Therefore, studying the distribution of organelles at the single-cell level is important to understand cellular function. Mesenchymal stem cells are multipotent cells that have been explored as a therapeutic method for treating a variety of diseases. Studying how organelles are structured in these cells can answer questions about their characteristics and potential. Herein, rapid multiplexed immunofluorescence (RapMIF) was performed to understand the spatial organization of 10 organelle proteins and the interactions between them in the bone marrow (BM) and umbilical cord (UC) mesenchymal stem cells (MSCs). Spatial correlations, colocalization, clustering, statistical tests, texture, and morphological analyses were conducted at the single cell level, shedding light onto the interrelations between the organelles and comparisons of the two MSC subtypes. Such analytics toolsets indicated that UC MSCs exhibited higher organelle expression and spatially spread distribution of mitochondria accompanied by several other organelles compared to BM MSCs. This data-driven single-cell approach provided by rapid subcellular proteomic imaging enables personalized stem cell therapeutics.

## Introduction

Cells perform different functions like providing structure and support, facilitating growth, producing energy, etc. to support and sustain life. These activities are handled by various subcellular structures termed organelles such as the nucleus, mitochondria, endoplasmic reticulum, and Golgi apparatus^1^. Organelles cooperate to form a network of interactions that enable different cellular activities^2^. Therefore, studying organelle interactions is key to understanding how cells function more comprehensively. Cell-to-cell variability is observed not only between cell types but also between cells of the same type, resulting in molecularly and functionally distinct cells^3^. Such differences may contribute to the health and function of the entire organism. Multiple factors, such as microenvironment variability, differences in the cellular stages, genetics or epigenetics, or fluctuations in gene expression levels, can cause this heterogeneity. Single-cell analysis approaches are thus useful in investigating aspects of cellular mechanisms that are not revealed in bulk-level studies^4,5^.

The use of mesenchymal stem cells (MSCs) has become a promising therapeutic method for treating a variety of diseases, as they can repair damaged cells by differentiating into replacement cells and modulating immune responses^6–11^. Therefore, analyzing the spatial organelle networks within MSCs can lead to a better understanding of cell functions to design appropriate treatment methods^12–16^. Although there have been recent studies focusing on spatial organelle analysis, these studies have used spatial data of each organelle from different cells, which reduces the accuracy of the resulting spatial information^17^. As such, a highly multiplexed protein imaging and analysis method is needed to obtain spatial information on organelles within the same cell, which can then be used to compare and understand differences in spatial organization between different cell types. In addition to intra-population variability, the most commonly used^18^, readily available stem cells sourced from bone marrow (BM) and postnatal umbilical cord (UC) introduce additional variables for the study. These cells display different molecular profiles, differentiation potential, and therapeutic efficacy^19^, and there is little consensus on how much, if any, the effect that MSC source has on outcome^20^.

Highly multiplexed imaging technologies enable the detection of multiple proteins in a single cell. Imaging mass cytometry (IMC)^21^ and multiplexed ion beam imaging (MIBI)^22^ can image up to 36 proteins using isotope-labeled antibody libraries and specialized equipment. However, these measurements are limited by their low resolution of about 0.5 to 1-μm. High-dimensional fluorescence imaging methods can map up to 50 proteins and include DNA-barcoded co-detection by indexing (CODEX) imaging^23^, multiplexed immunofluorescence microscopy (MxIF)^24^, and cyclic and sequential IF techniques^25–28^. While conventional immunofluorescence (IF) is time-consuming, rapid multiplexed immunofluorescence (RapMIF) provides multiplicity through quick multiple rounds of immunostaining and fluorophore inactivation. RapMIF enables high-throughput in situ proteomic analysis using conventional microscopes.

Herein, we established a rapid protein analysis pipeline for deciphering spatial organelle networks within a single cell. To achieve this, proteins in key organelles in mesenchymal stem cells (MSCs) such as the nucleus, mitochondria, Golgi, and endoplasmic reticulum (ER) have been targeted using antibodies. Multiplexed protein imaging was performed to examine the spatial organization of organelles and the interactions between organelles in MSCs. The analysis of organelle interactions on a single-cell level can eventually aid the stem-cell field in better understanding cell functions and exploring treatment methods using MSCs. Differences in organelle localization, interactions, and associated energy highlight the MSC heterogeneity from donor sources that will better inform therapeutic cell designs.

## Results

### Multiplexed protein labeling reveals spatially resolved subcellular organelle maps in tissue-specific MSCs

**Figure 1:**
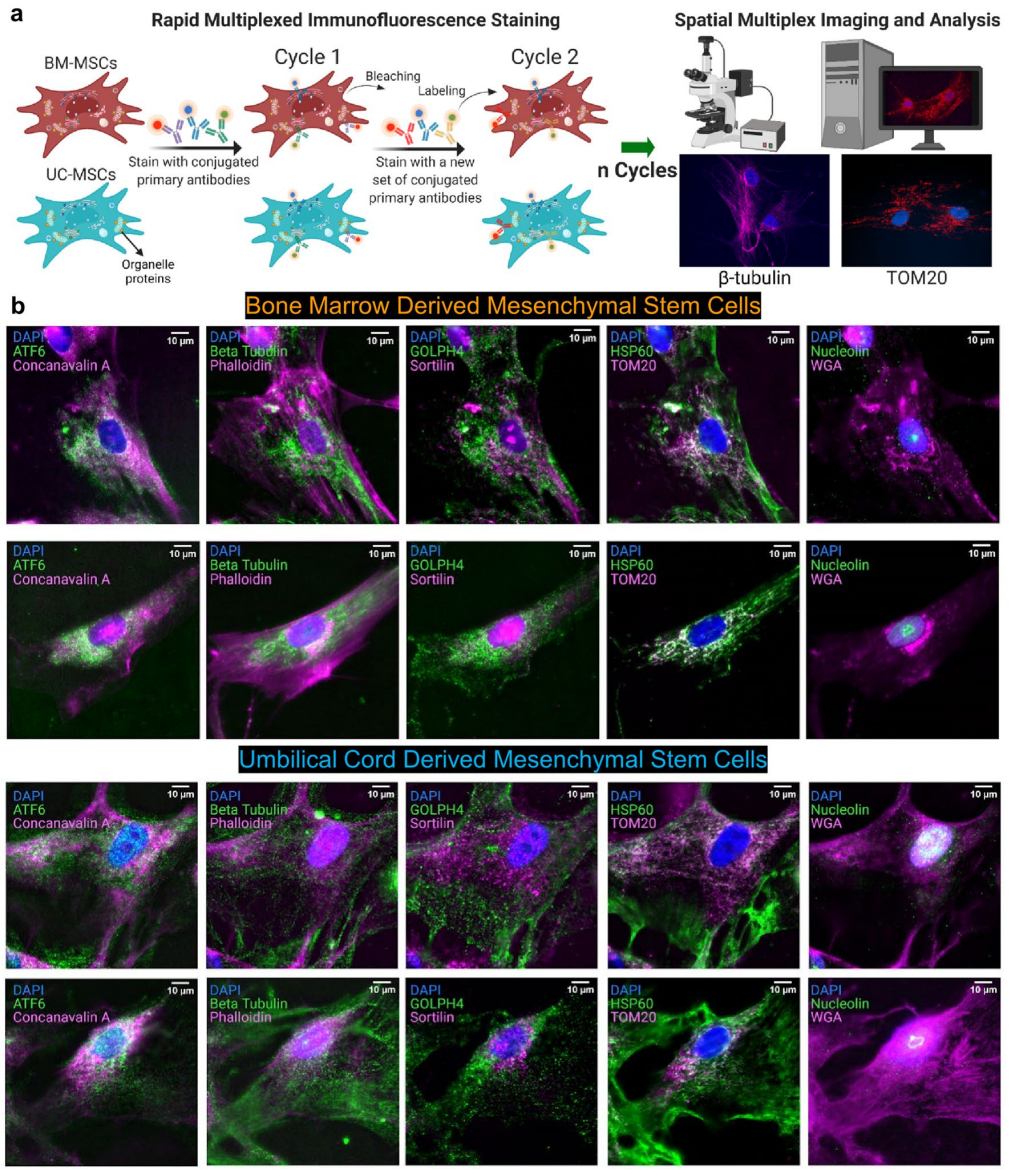
Ten-plex organelle mapping in mesenchymal stem cells using rapid multiplexed immunofluorescence (RapMIF). (**a**) Schematic of RapMIF for organelle analysis in MSCs. Each cycle contains 3 conjugated antibodies plus 4′,6-diamidino-2-phenylindole (DAPI) followed by bleaching of the signal before the next cycle consisting of 3 new antibodies. Imaging of the 3 antibodies before and after bleaching confirms the presence of signal and then signal removal. Multiplex imaging consists of multiple cycles (n) of antibody labeling and bleaching. BM MSCs (brown) and UC MSCs (cyan) are labeled with the same multiplex antibodies that target the same set of organelles. All images are acquired on Nikon widefield and registered across cycles to produce a final set of multiplex-labeled images. Example images show Beta Tubulin (magenta, left) and TOM20 (red, right) overlaid with the nucleus in DAPI (blue). Created with BioRender.com. (**b**) Visualization of organelle markers in single cells from BM MSCs and UC MSCs. Each row corresponds to a distinct single cell. The top 2 rows show BM MSCs and the bottom 2 rows show UC MSCs. Multiplexed markers for the same cell are displayed across 4 columns (Column 1: ATF6 & Concanavalin A, Column 2: Beta Tubulin & Phalloidin, Column 3: GOLPH4 & Sortilin, Column 4: HSP60 & TOM20, Column 5: Nucleolin & WGA). Each image displays DAPI with a pair of organelle markers in magenta and green. DAPI is used to register the signals across cycles. Signal removal is confirmed with a widefield microscope after bleaching each cycle. All scale bars 10 µm.

To measure single-cell organelle distributions, we profiled subcellular localization of organelle proteins in the bone marrow and umbilical cord MSCs using RapMIF (Fig. 1a, Supplementary Fig. 1a). Multiple markers colocalized in different cellular regions, including mitochondria (TOM20 and HSP60), the Golgi (Sortilin, GOLPH4, and Wheat Germ Agglutinin: WGA), endoplasmic reticulum (ATF6 and Concanavalin A), nucleolus (Nucleolin), microtubules (β-Tubulin), and actin filaments (Phalloidin) (Fig. 1b, Supplementary Figs. 1b and 2). These colocalizations were quantified using scatter plots, correlation coefficients, clustering, and texture analysis to understand the spatial organization of these markers and the interactions between them.

**Figure 2:**
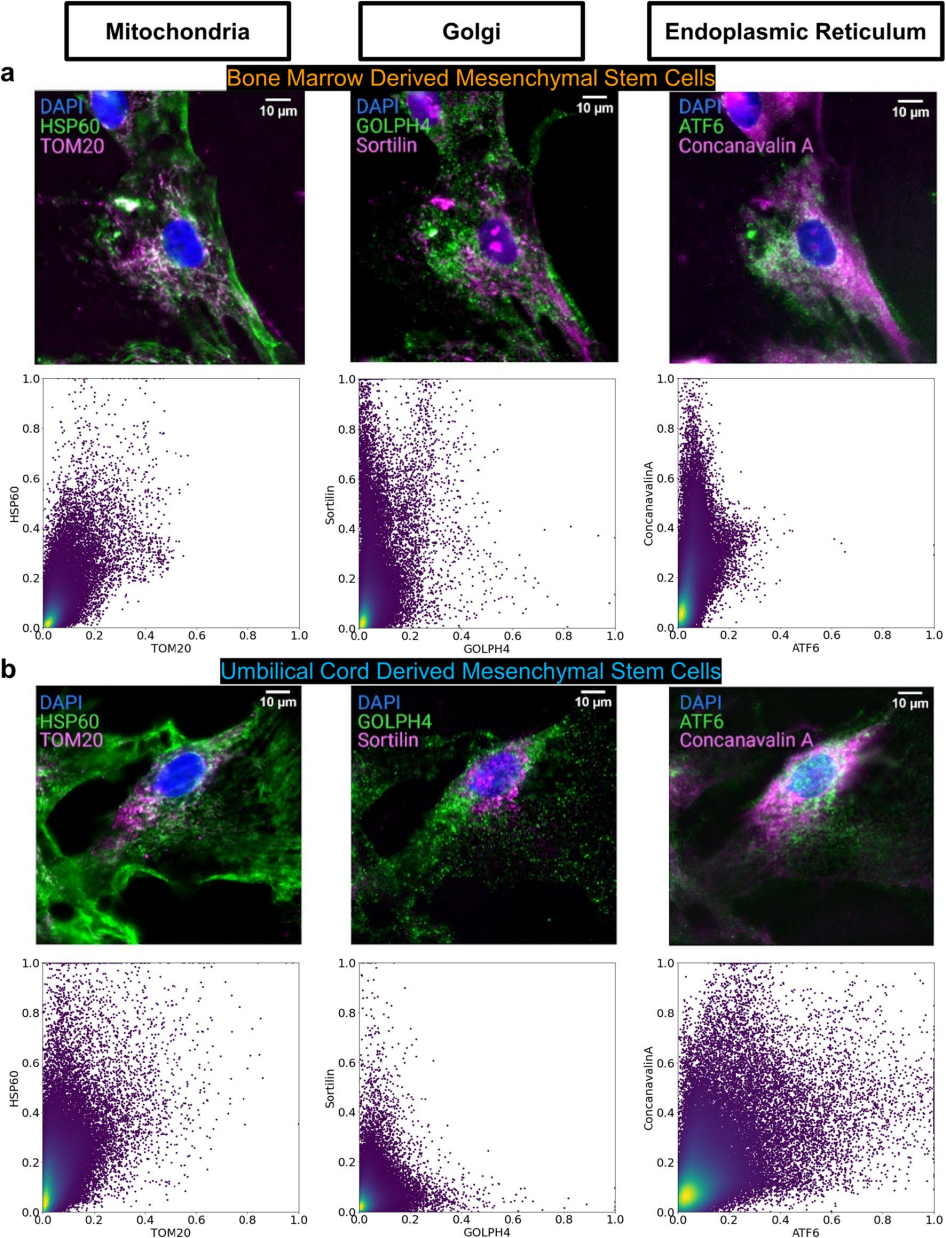
Comparison of protein markers targeting the same organelles in BM MSCs and UC MSCs. (**a**) Single BM MSC example with various organelles across the columns. Each organelle is shown by a marker pair: HSP60 & TOM20 (mitochondria, left), GOLPH4 & Sortilin (Golgi, middle), ATF6 & Concanavalin A (ER, right). All scale bars 10 µm. The bottom row shows intensity scatter plots with 50,000 pixels for each marker pair colocalized within the same organelle. The x and y-axis indicate the min–max scaled intensity values of the markers. More colocalized pixels appear closer to the y = x diagonal while less colocalized pixels appear closer to either axis, belonging more to that particular marker. In BM MSCs, mitochondria antibodies are more colocalized than either Golgi or ER. (**b**) Single UC MSC example with various organelles across the columns. Each organelle is shown by a marker pair: HSP60 & TOM20 (mitochondria, left), GOLPH4 & Sortilin (Golgi, middle), ATF6 & Concanavalin A (ER, right). All scale bars 10 µm. The bottom row shows intensity scatter plots with 50,000 pixels for each marker pair colocalized within the same organelle. The x and y-axis indicate the min–max scaled intensity values of the markers. More colocalized pixels appear closer to the y = x diagonal while less colocalized pixels appear closer to either axis, belonging more to that particular marker. In UC MSCs, ER antibodies are more colocalized than either mitochondria or Golgi.

From the 10-plex data, up to 25 cells (BM and UC MSCs combined) were selected for each marker. The scatter plots of intensity were calculated by random sampling of 50,000 pixel intensity values from all the cells for each marker pair targeting the same organelle. The scatter plots showed similar distribution for BM and UC MSCs for mitochondria targeting markers, implying similar interactions between the mitochondria markers in the two cell types (Fig. 2). However, the point distribution difference in scatter plots for Golgi and ER markers between BM and UC MSCs can be attributed to differences in interactions and the subcellular targets of the markers in the two cell types.

### UC MSC organelles exhibit higher protein expression over a larger spatial area

**Figure 3:**
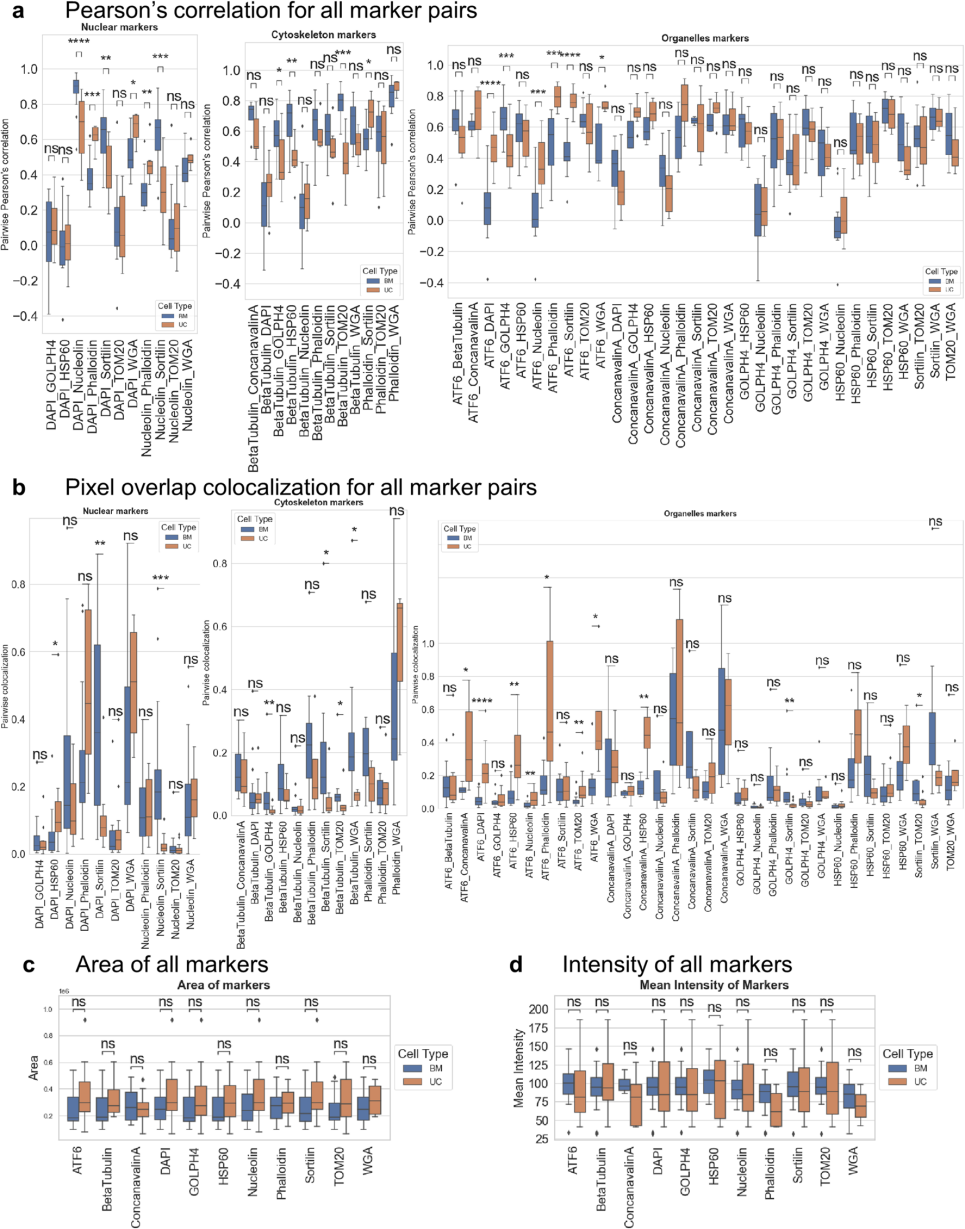
Spatial analysis of organelle colocalization in BM MSCs and UC MSCs. (**a**) Boxplots of Pearson’s correlation of marker pairs per cell between all BM MSCs (blue) and UC MSCs (orange) categorized by nuclear, cytoskeleton, and organelle markers. Box plots show the median, first and third quartile, minimum, and maximum (excluding outliers). The outliers are marked as individual points. Stars denote the statistical significance for pairwise comparison. *p* value was calculated using the Mann–Whitney test (ns: *p* >  = 0.05, ****p <  = 0.0001). BM MSCs and UC MSCs exhibit the greatest differences in colocalized expression among pairs that include mitochondria (TOM20, HSP60), cytoskeleton (Beta Tubulin, Phalloidin), and endoplasmic reticulum (Concanavalin A). **(b)** Boxplots of pixel overlap colocalization values of marker pairs per cell between all BM MSCs (blue) and UC MSCs (orange) categorized by nuclear, cytoskeleton, and organelle markers. Box plots show the median, first and third quartile, minimum, and maximum (excluding outliers). The outliers are marked as individual points. Stars denote the statistical significance for pairwise comparison. *p* value was calculated using the Mann–Whitney test (ns: *p* >  = 0.05, ****p <  = 0.0001). UC MSC markers express a higher fraction of colocalized pixels in ER (ATF6, Concanavalin A) and mitochondria (HSP60) than those of BM MSCs, suggesting more active crosstalk of organelles in UC MSCs. **(c)** Comparison of the total area of 11 markers per cell between all BM MSCs (blue) and UC MSCs (orange) using boxplots. Box plots show the median, first and third quartile, minimum, and maximum (excluding outliers). The outliers are marked as individual points. Stars denote the statistical significance for pairwise comparison. *p* value was calculated using the Mann–Whitney test (ns: *p* >  = 0.05, ****p <  = 0.0001). UC MSC markers generally express larger and more variable areas than those of BM MSCs, suggesting that these organelles serve a higher energetic role in UC MSCs. **(d)** Comparison of the total intensity of 11 markers per cell between all BM MSCs (blue) and UC MSCs (orange) using boxplots. Box plots show the median, first and third quartile, minimum, and maximum (excluding outliers). The outliers are marked as individual points. Stars denote the statistical significance for pairwise comparison. *p* value was calculated using the Mann–Whitney test (ns: *p* >  = 0.05, ****p <  = 0.0001. Markers in UC MSCs express a larger range of intensities than in BM MSCs and thus these UC MSC organelles are in a more active state.

To quantify organelle interrelation patterns, pairwise Pearson’s correlation and pixel overlap colocalization values were obtained for each unique marker pair and plotted as boxplots with the Mann–Whitney test to observe significance (*p* < 0.05) (Fig. 3a, b, Supplementary Tables 1, 2, 3, and 4). Some marker pairs consistently showed significance in both the plots (ATF6_DAPI, β-Tubulin_GOLPH4, and β-Tubulin_TOM20) indicating a difference in colocalization between BM and UC MSCs. In general, marker pairs containing ATF6, β-Tubulin, DAPI, GOLPH4, and HSP60 expressed significant differences in distribution between the two cell types. Overall, UC MSCs showed more colocalization between ER (ATF6, Concanavalin A) and mitochondria (HSP60 TOM20) pairs, suggesting more crosstalk between their organelles. Other than the pairwise plots, the area and average intensity values of each marker were obtained per cell and compared between BM and UC MSCs (Fig. 3c, d, Supplementary Tables 5 and 6). While the distribution of area and intensity exhibited weak significant *p* values for any marker, UC MSC organelles, in general, exhibited higher protein expression for organelle markers and larger mean area per marker. The median area of UC MSCs was higher than the median area of BM MSCs except for Concanavalin A. The range of average intensity values for UC MSCs was higher than the range for BM MSCs. This could be due to higher cell-to-cell variability in intensity values for UC MSCs compared to BM MSCs.

### Organelles share distinct pixel overlap colocalizations within single BM and UC MSCs

Because BM and UC MSCs expressed different spatial organelle patterns, we reasoned these differences could be recapitulated in their single-cell distributions. Single-cell analysis and comparisons were performed by selecting 4 organelle protein markers (ATF6, GOLPH4, Nucleolin, and TOM20) for 7 BM and UC MSCs. DAPI was used as an additional marker to locate the nucleus. These markers were selected based on their specificity to the target organelle (Supplementary Table 1). Phalloidin, Concanavalin A, and WGA, while highlighting organelles and cellular components, also work as cell segmentation markers, and are therefore less specific^29^. While TOM20 and HSP60 both colocalize in mitochondria, HSP60 can also be found in extramitochondrial regions such as the cytosol, vesicles, and the cell membrane^30–32^. Similarly, Sortilin can be found in regions other than the Golgi, such as endosomes and lysosomes, whereas GOLPH4 is predominantly localized in the Golgi^33,34^.

**Figure 4:**
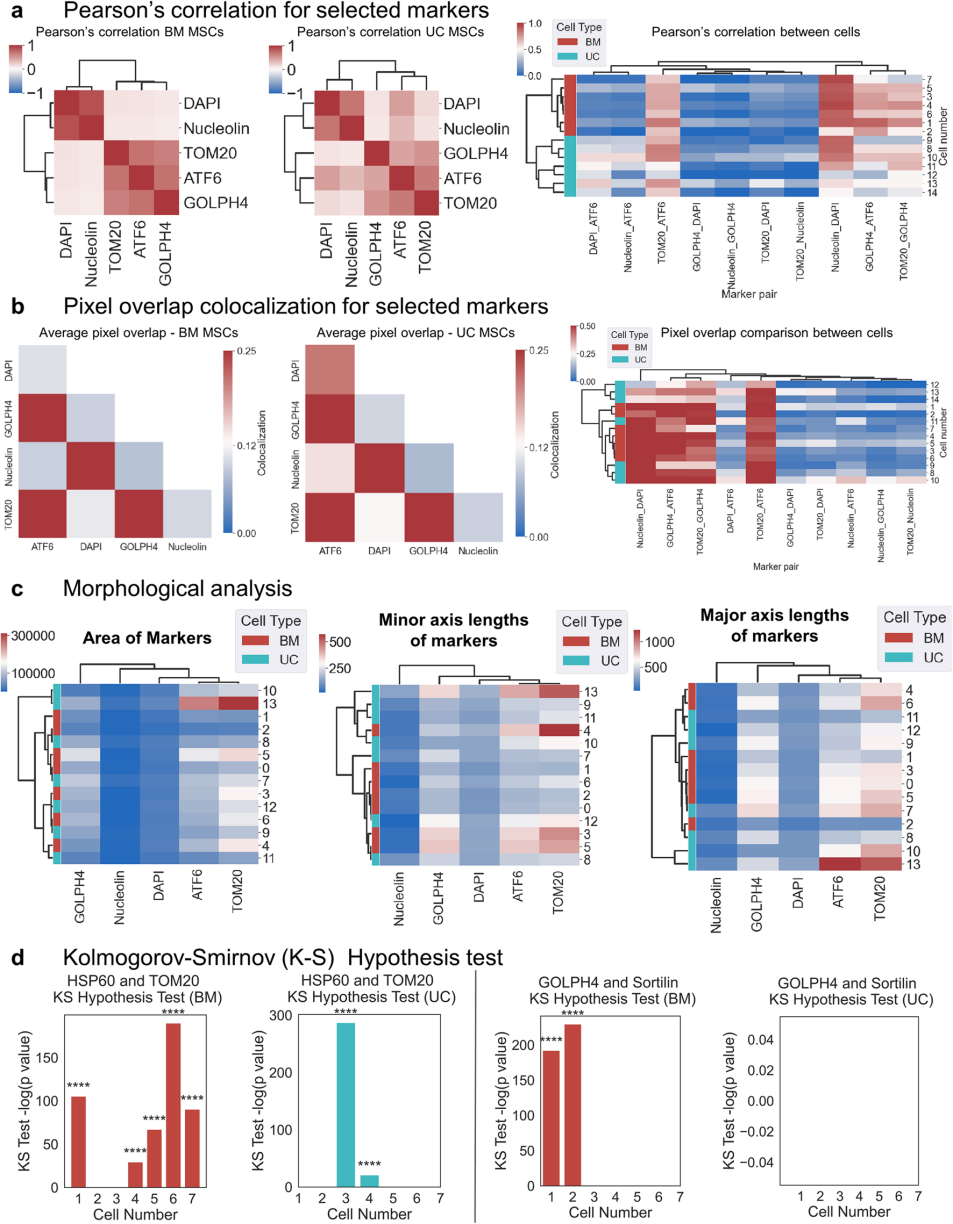
Multiplexed protein analysis of organelle markers in BM MSCs and UC MSCs. (**a**) Average Pearson’s correlation of the total intensity of 5 markers in BM MSCs and UC MSCs per marker per cell plotted as a heatmap. BM MSCs are shown on the left, UC MSCs are shown in the middle, and combined Pearson’s for each cell is shown on the right. The comparison of the correlation of 5 markers between BM MSCs (n = 7) and UC MSCs (n = 7) was provided as a heatmap using the average linkage method based on the correlation distance. Larger correlation values are shown in red and smaller correlation values are shown in blue. BM MSCs possess more separation between nuclear and cytosolic organelles while UC MSCs are less separated, which illustrates that UC MSC organelles are more spread across the cell. The right side shows the single-cell heatmap for all marker pairs. UC MSCs exhibit higher correlations in DAPI_ATF6, TOM20_ATF6, and Nucleolin_ATF6, suggesting more crosstalk between nuclear and cytosolic organelles. Single BM MSCs exhibit stronger correlations within nuclear or cytosol pairs e.g. Nucleolin_DAPI, GOLPH4_ATF6, TOM20_GOLPH4, implying that nucleus and cytosol organelles are more segregated in BM MSCs, which agrees with the left heatmap. **(b)** Average pixel overlap colocalization between 5 markers in BM MSCs and UC MSCs per marker per cell plotted as a heatmap. The comparison of pixel overlap between BM MSCs (left) and UC MSCs (middle) was provided as a heatmap using the average linkage method based on the contact frequency distance. The combined pixel overlap on a single cell level is shown on the right. Large pixel overlap values are shown in red and small pixel overlap values are shown in blue. UC MSCs possess slightly higher pixel overlap values among organelles in different compartments (nuclear vs cytoskeleton) but this difference is less pronounced than in **(a)**. A few UC MSCs (10, 12, 13, 14) show distinct patterns from other UC MSCs in terms of weaker pixel overlap in Nucleolin_DAPI, GOLPH4_ATF6, TOM20_GOLPH4, Nucleolin_ATF6, TOM20_Nucleolin. UC MSCs exhibit higher variability in pixel overlap. **(c)** Heatmaps were generated to compare the morphological features and to determine any close relationships or associations between markers and between BM MSCs (red) and UC MSCs (teal). Morphology is defined as area (left), minor axis (middle), and major axis (right). Both cell types exhibit more differences in minor and major axes and fewer differences in terms of area, implying that organelles differ more in shape and less in expression area. The most notable difference is TOM20, suggesting a difference in mitochondrial energetic activity. Most differences, such as TOM20 and ATF6, are attributable to UC MSCs, suggesting more single-cell variability among UC MSCs. **(d)** Kolmogorov–Smirnov (K–S) hypothesis test was conducted between organelle marker pairs targeting the mitochondria (TOM20 and HSP60; left) and Golgi (GOLPH4 and Sortilin; right) to study if similar proteins express different spatial distributions within each cell. Single BM MSCs are shown in red while single UC MSCs are shown in teal. In terms of spatial mitochondrial expression, BM MSCs have more single-cell variability while UC MSCs express more uniformly. BM MSCs possess more single-cell variability than UC MSCs concerning spatial ER expression.

To explore how organelles colocalized in subcellular regions, we used two metrics, including Pearson’s correlation and pixel overlap colocalization. Pearson's correlation coefficients between the selected markers for each cell were calculated, and the average values for BM and UC MSCs were plotted as a heatmap with dendrograms (Fig. 4a, Supplementary Figs. 3 and 4, Supplementary Table 7). The elements of the correlation heatmaps denote the location concordance between the nuclear and cytosolic markers^17,35^. ATF6 has a higher correlation with nuclear markers in UC MSCs than in BM MSCs. On the other hand, GOLPH4 has a slightly lower correlation with ATF6 and TOM20 in UC cells compared to the BM cells. In general, UC MSCs exhibit higher spatial correlation than BM MSCs between nucleus and cytosol, suggesting that UC MSC organelles are spread over a larger area, agreeing well with Fig. 3c, and are thus considered as energetically more active cells in their function. To study the single-cell variation in correlation coefficients in the cells, the pairwise correlation coefficients were plotted as a heatmap across all cells (Fig. 4a, right). The heatmap shows the cell-to-cell variation in the correlation coefficients between marker pairs and also indicates the differences between BM and UC cells. Some markers exhibit higher cell-to-cell variability. The correlation coefficients between marker pairs Nucleolin_DAPI, GOLPH4_ATF6, TOM20_GOLPH4, and TOM20_ATF6 are higher than other marker pairs for most of the cells. These pairs also have a higher correlation in BM than UC MSCs, revealing that nuclear pairs and cytosol pairs are more separated in BM than in UC MSCs. Likewise, the pairwise correlation coefficients for Nucleolin_ATF6 and DAPI_ATF6 are higher in UC MSCs compared to BM MSCs, indicating a higher interaction between the nucleus and ER in UC MSCs. Interestingly, the same trend is less prominent for interaction between the nucleus and mitochondria (e.g. DAPI_TOM20, Nucleolin_TOM20). These differences in correlation values suggest that the UC MSCs possess more prevalent proteomic activity in their organelles than BM MSCs.

Pixel overlap colocalization, *i.e.*, the metric that counts the number of overlapping pixels after thresholding normalized to the cell area, was another method to measure colocalization between the markers for each cell. These average values were then plotted as a heatmap (Fig. 4b, Supplementary Figs. 5 and 6, Supplementary Table 8). This parameter is similar to Mander's coefficients, except that it gives an absolute number of pixels that are overlapping between the two images^36^. Similar to Pearson's correlation, UC MSCs show higher pixel overlaps than BM MSCs, especially between DAPI and ATF6, again suggesting more crosstalk between the nucleus and ER in UC MSCs. The colocalization for BM MSCs and UC MSCs was combined and plotted as another heatmap to compare the cell-to-cell differences (Fig. 4b, right). A few UC MSCs express marker pairs differently than the rest of the cells, suggesting more single-cell variability in that population. Among the organelle markers, there is higher pixel overlap colocalization in Nucleolin_DAPI, GOLPH4_ATF6, TOM20_GOLPH4, and TOM20_ATF6, implying fewer interactions between nucleus and cytosol except for a few UC MSCs: 10, 12, 13, and 14. The higher single cell variability in UC MSCs is consistent with the observation from Pearson’s correlation coefficient. The pixel overlap colocalization between cytosolic markers and nuclear markers was expectedly low, which was also observed in Pearson’s correlation coefficient.

### Spatial spread over major and minor axes of organelles distinguishes UC and BM MSCs better than area only

In addition to colocalization differences, we hypothesized that there are considerable differences in morphology and size between UC and BM MSCs. The heatmaps of different markers’ areas indicate that UC MSCs and BM MSCs show more differences along major and minor axes than area (Fig. 4c, Supplementary Table 9), suggesting that organelles are expressed in different morphology and shapes rather than varying in area. This is especially crucial with TOM20, which suggests a stark difference in mitochondrial energetic activity. ATF6 and TOM20 have similar areas, while GOLPH4 has a smaller area by comparison. The nuclear markers are smaller in comparison to the cytosolic markers. The major and minor axis heatmaps show a better distinction between BM MSCs and UC MSCs (Fig. 4c, Supplementary Tables 10 and 11). As expected, the markers found in the nucleus, DAPI, and Nucleolin have smaller major and minor axis values across all cells. TOM20 has the largest major and minor axis values, followed by ATF6 and then GOLPH4. Differences in spatial spreading in TOM20 and ATF6 expression are attributable to more UC than BM MSCs. The mitochondria being larger than the ER also suggests a more energetically active state for these UC MSCs.

### UC-MSCs display consistently higher mitochondrial expression than BM-MSCs

To statistically benchmark how differently single cells express the same organelle, we implemented the Kolmogorov–Smirnov (K–S) hypothesis test to highlight significant differences between the spatial distribution of organelle marker pairs, including GOLPH4/Sortilin and TOM20/HSP60 (Fig. 4d, Supplementary Table 12). Concerning their spatial distributions around the cell’s center of mass, GOLPH4 and Sortilin, which both target the Golgi apparatus, express similar patterns within most BM and UC MSCs. On the other hand, spatial distributions concerning the cell’s center of mass for HSP60 and TOM20, which both target the mitochondria, exhibit different spatial patterns within more BM MSCs than UC MSCs (Fig. 4d). A few cells exhibit moderate or no levels of significance between HSP60 and TOM20 spatial expression. Between the two cell types, BM MSCs show a higher amount of significant differences between the spatial distributions of the markers compared to UC MSCs, indicating higher single-cell variability in the expression of markers. Since UC MSCs exhibit larger HSP60 and TOM20 areas (Fig. 3c) with more variable intensities (Fig. 3d), we conclude that UC MSCs are more uniformly expressing higher mitochondrial activity, and, thus, UC MSCs are more energetic than BM MSCs.

### Multiplexed pixel clustering indicates cell-type specific organelle interactions

**Figure 5:**
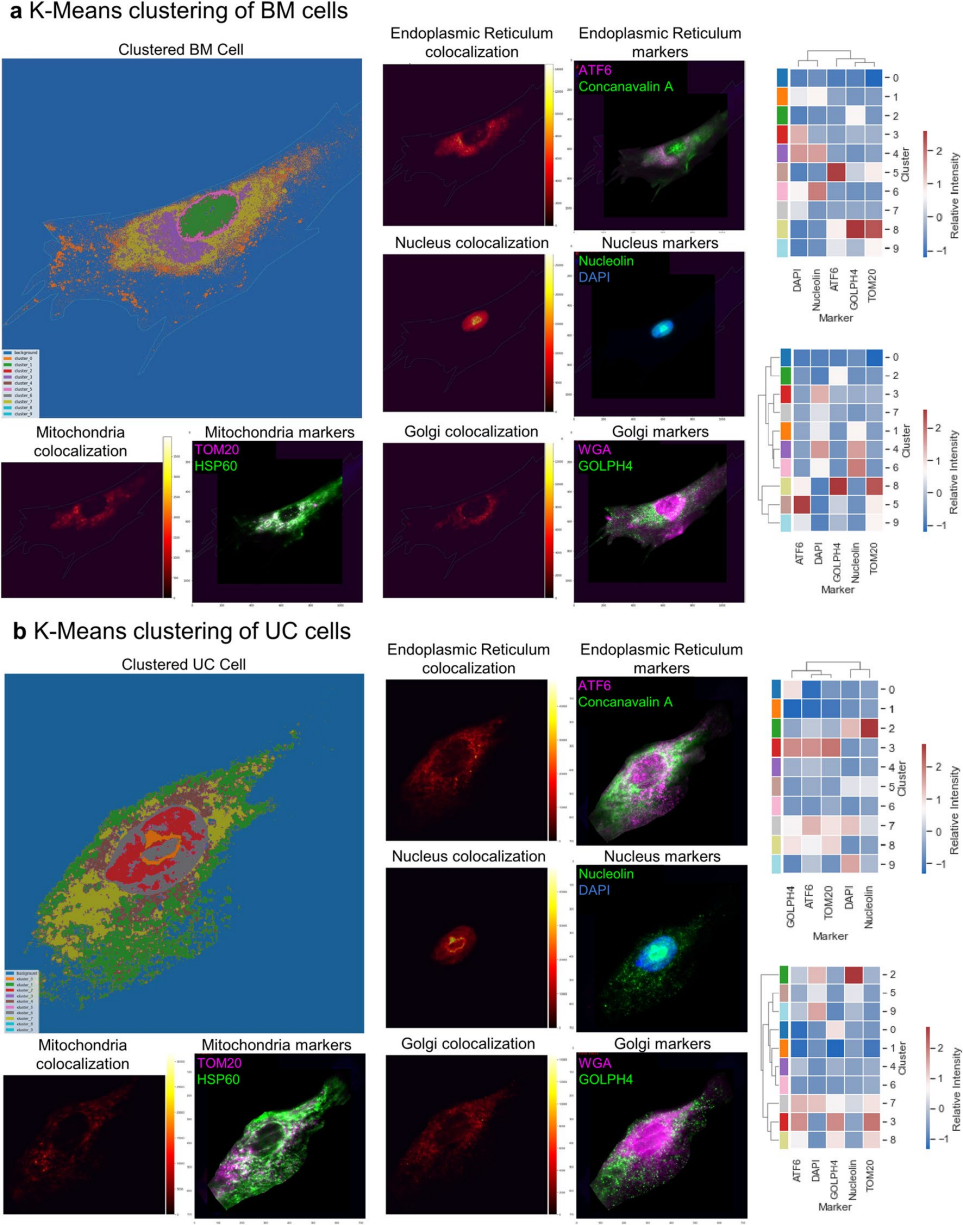
Pixel level clustering of organelle markers in BM MSCs and UC MSCs. (**a**) K-Means clustering of intensities of markers for a single BM MSC. 10 clusters were chosen and colored back on the original cell (large left). Each cluster represents one distinct expression profile of the protein markers in the cells. A pair of images are shown for each organelle (mitochondria, Golgi, Nucleus, ER): (1) an overlay of the two markers that target the organelle (right) and (2) the colocalization of the two markers obtained by multiplying the two marker images pixel-wise from the right to highlight areas of overlap (left). Example: ATF6 and Concanavalin A forming the ER localization. Yellow values indicate areas of higher overlap while red values indicate areas of lower overlap. The right side shows the heatmap of marker intensity with a dendrogram based on marker intensities (right, top) and clustering results (right, bottom). From the dendrogram based on marker intensities, GOLPH4 and TOM20 are directly linked, along with ATF6. This suggests moderate crosstalk between ER and mitochondria, especially in cluster 8. Nuclear organelles DAPI and Nucleolin are associated in a separate cluster. (**b**) K-Means clustering of intensities of markers for a single UC MSC. 10 clusters were chosen and colored back on the original cell (large left). Each cluster represents one distinct expression profile of the protein markers in the cells. A pair of images are shown for each organelle (mitochondria, Golgi, Nucleus, ER): (1) an overlay of the two markers that target the organelle (right) and (2) the colocalization of the two markers obtained by multiplying the two marker images pixel-wise from the right to highlight areas of overlap (left). Example: ATF6 and Concanavalin A forming the ER localization. Yellow values indicate areas of higher overlap while red values indicate areas of lower overlap. The right side shows the heatmap of marker intensity with a dendrogram based on marker intensities (right, top) and clustering results (right, bottom). From the dendrogram based on marker intensities, ATF6 and TOM20 exhibit moderate crosstalk (clusters 3, 7, 8) but not as intensely as the BM MSC in (**a**).

We asked whether there are more organelle interactions beyond colocalized nucleus and cytosol or not. To uncover this puzzling question about organelle interactions, unsupervised, pixel-level clustering was performed on the dataset consisting of intensity values of all the markers^26^. The goal of performing pixel-level clustering is to identify a subset of pixels that share a unique multiplexed intensity profile. These clusters could indicate an organelle pattern or regions of similar functionality, and their hierarchical relationship reveals communication among them. The K-Means clustering algorithm was used to group the pixels into 10 clusters. The two cell types were clustered independently, and the resultant clusters were colored back on the images of the single cells. The spatial mapping of a single cell’s clustered regions was compared with the organelle colocalization calculated as the product of markers targeting that organelle (Fig. 5, left). The pixel-level clustering algorithm highlighted certain organelle patterns, notably showing a distinction between the nucleus and cytosol. The organelle marker distributions of each cluster were plotted as cluster maps (Fig. 5, right). The dendrograms show different hierarchical clustering patterns and cluster intensities across markers for BM and UC cells. TOM20 is directly associated with ATF6 or GOLPH4 in both UC and BM MSCs, suggesting crosstalk between mitochondria and ER (Fig. 5a,b, right). DAPI and Nucleoli are also directly linked in both of these cell types, implying segregation of nuclear interaction before reaching the cytosol. The clustering results also indicate a stronger association of GOLPH4 and TOM20 in BM than in UC MSCs (Fig. 5, right bottom), which suggests more crosstalk between ER and mitochondria in BM MSCs. Given the potential higher energetic activity of UC MSCs observed in previous sections, the organelle energy is directed elsewhere than the ER in UC MSCs.

### Superpixel segmentation informs content-aware spatially distinct features of each organelle in UC and BM MSCs

**Figure 6:**
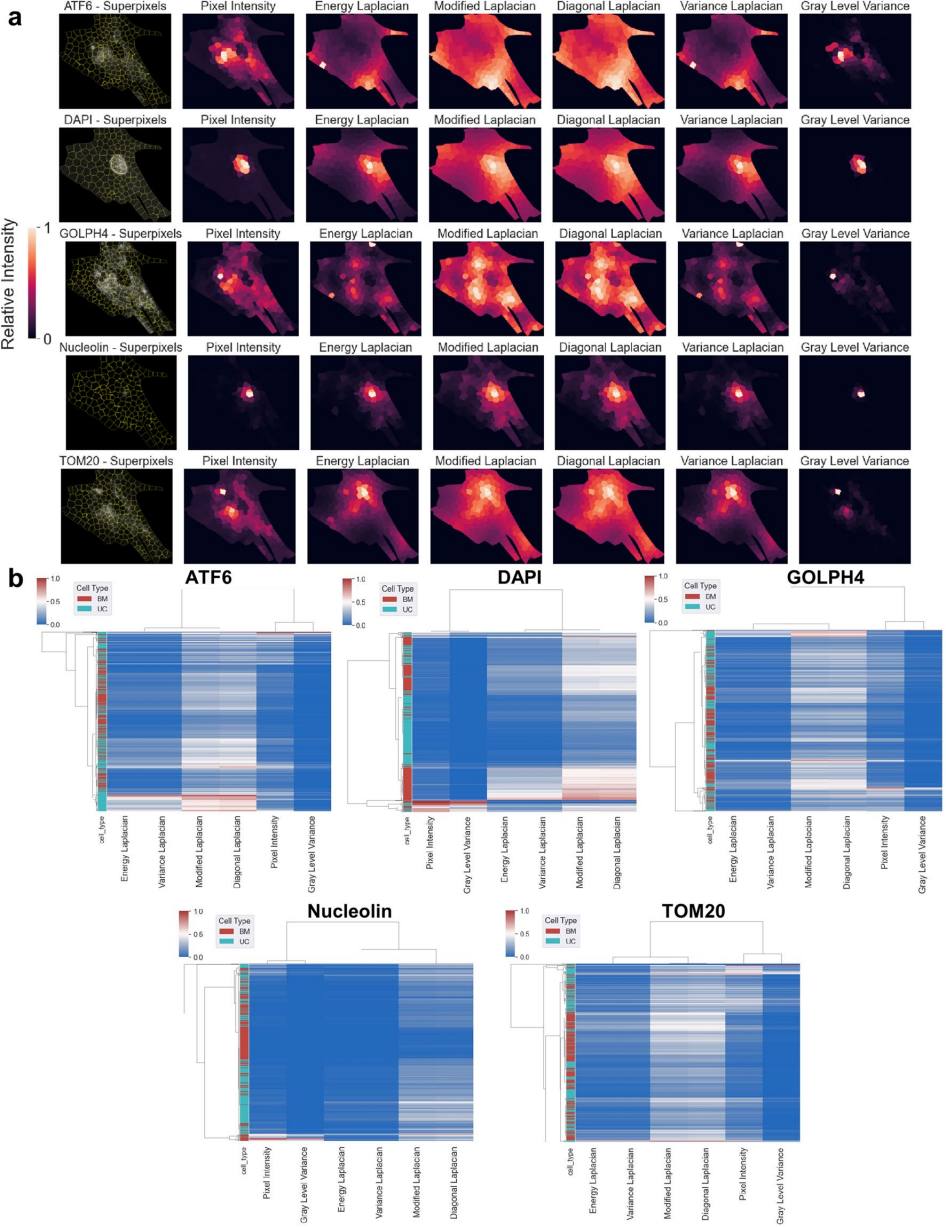
Superpixel segmentation and texture analysis of organelle markers in BM MSCs and UC MSCs. (**a**) Superpixel segmentation and texture features using various cost functions (Pixel Intensity, Energy Laplacian, Modified Laplacian, Diagonal Laplacian, Variance Laplacian, and Gray Level Variance) calculated on superpixels for each marker of a single BM MSC. Each column represents a different superpixel method. Segmentation identifies subcellular, regional hotspots of organelles. Modified Laplacian and diagonal Laplacian create a more spread signal for all markers. Pixel intensity and variance Laplacian reveal discontinuous hotspots of GOLPH4**,** ATF6, and TOM20. (**b**) Heatmap of texture feature values for each superpixel in 7 BM MSCs and 7 UC MSCs for each marker. Each row denotes a single superpixel, and the column contains texture feature values for the corresponding superpixel. The feature values range from 0 (blue) to 1 (red) in each heatmap. Modified Laplacian and diagonal Laplacian yield the largest feature values. Each heatmap represents a different marker. UC and BM MSCs show similar overall patterns across various superpixels, implying that earlier, quantified differences are more minute and superpixel methods disguise these small differences when downsampling.

Finally, to evaluate the spatially distinct features of our area and colocalization results (Fig. 4c), we analyzed several organelles with a superpixel segmentation approach using K-Means^37^. In this technique, the content-aware texture features were calculated to observe the spatial patterns of each marker. The various texture features used pixel intensity, energy Laplacian, modified Laplacian, diagonal Laplacian, variance Laplacian, and gray level variance. The images were segmented into superpixels by grouping the pixels around dense organelle regions across several subcellular partitions (n = 250), and the different texture features were calculated for each marker (Fig. 6a). The texture feature plots highlight the variations in the spatial organization of the marker. The superpixels containing the highest texture features indicate the region of maximum colocalization for each marker. Heatmaps of the texture features were obtained for each marker to compare the spatial patterns between BM and UC MSCs (Fig. 6b). The heatmaps indicate some similarities in texture features within BM and UC MSC superpixels. ATF6, DAPI, GOLPH4, and TOM20 in particular show higher similarity within each cell type, based on the number of superpixels clustered together. For nuclear markers, pixel intensity, gray level variance, and variance Laplacian illustrated clearer regions than the other methods (Fig. 6a). Modified and diagonal Laplacian features produced more spread results for all the markers, and are thus less informative. Interestingly, GOLPH4 and ATF6 were shown to localize in different hotspots around the cytosol as seen with energy Laplacian and variance Laplacian. This spread explains its colocalization with mitochondria seen in previous figures because a more discontinuous, spread organelle has a higher likelihood of colocalizing with other organelles in the cytosol. Quantification of texture features demonstrated that modified and diagonal Laplacians yield higher superpixel values, especially in UC MSCs (Fig. 6b), yielding additional content-aware spatial features to complement the previously discussed higher energetic activity of UC MSCs compared to BM MSCs.

### Virtual reality enables interactive visualization and quantification of spatial organelle maps of MSCs

**Figure 7:**
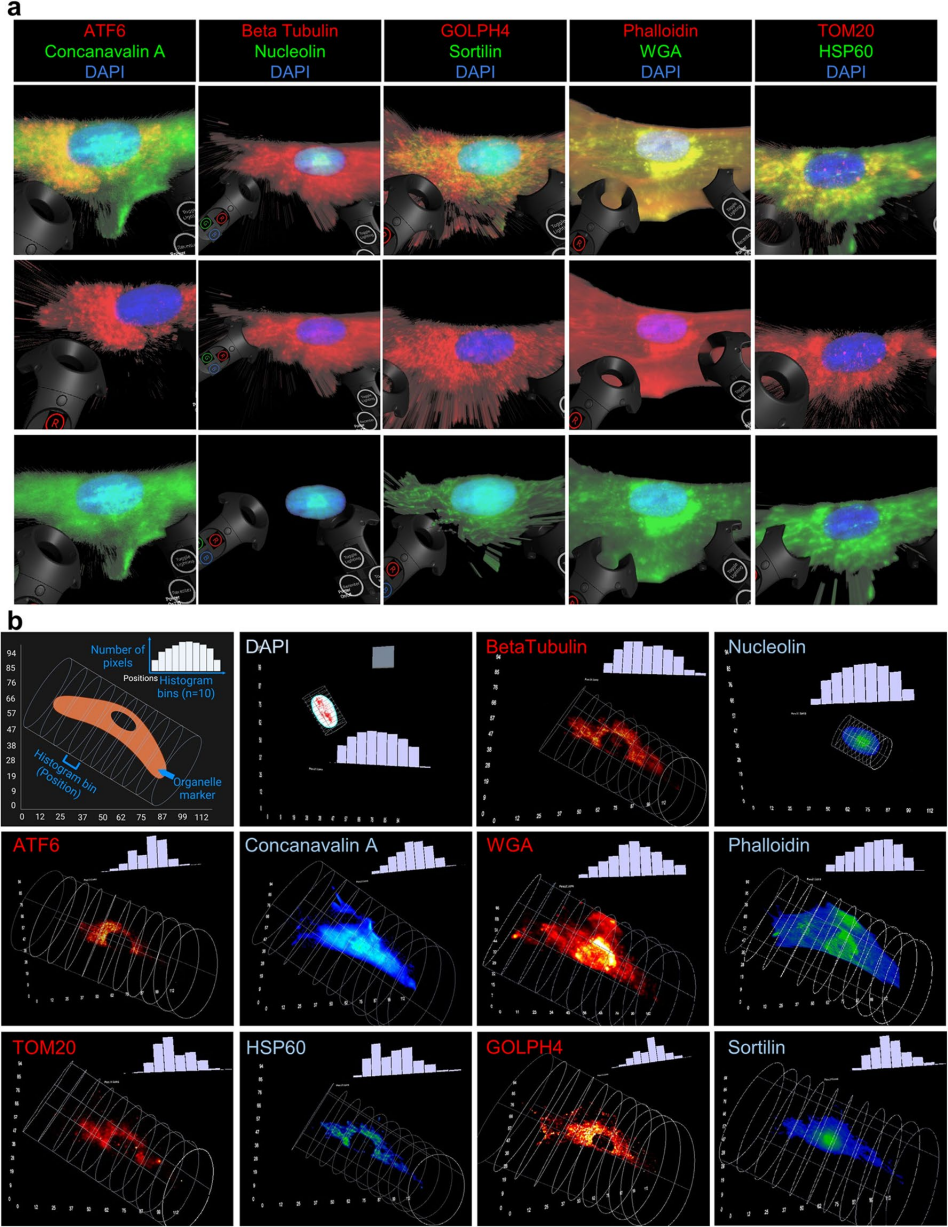
Virtual reality-based visualization and analysis of organelle imaging data in MSCs. (**a**) Visualization of organelle protein images (10 markers) for one BM MSC in ConfocalVR^62^. For each cell, the top shows the RGB image of the combined markers, while the bottom two show a single marker image in either red or green with DAPI. Different organelle markers are illustrated across the columns. Merged images are shown in the first row. Column 1: ATF6 (middle row) & Concanavalin A (bottom row), Column 2: Beta Tubulin (middle row) & Nucleolin (bottom row), Column 3: GOLPH4 (middle row) & Sortilin (bottom row), Column 4: Phalloidin (middle row) & WGA (bottom row), Column 5: TOM20 (middle row) & HSP60 (bottom row). Handset toggle switches used to interact with the image are shown on the left and right sides. (**b**) Visualization of organelle protein images (10 markers) for one BM MSC in Genuage^63^. The histogram of pixel count in 10 cylindrical bins (shown in white) of width 12.5 pixels (108 nm/pixel, bin width 1.354 µm) was plotted in the software. The top left image is an illustration explaining how cells are binned across the length to gauge spatial variability. In each box, red and blue colors illustrate the marker according to the upper left label. Beta Tubulin, ATF6, TOM20, and GOLPH4 show discontinuous regions of signal, supported by the uneven histograms, while the other signals are more continuous with more Gaussian histograms.

In addition to the open-source and user-based analysis of organelle data, the multiplexed proteomic images were interactively visualized in Virtual Reality (VR)^38^ platform using the software ConfocalVR and Genuage to visually explore our quantitative findings in an immersive experiential learning environment. In Confocal VR, organelle markers targeting the same organelle (Column 1: ATF6 & Concanavalin A, Column 2: Beta Tubulin & Nucleolin, Column 3: GOLPH4 & Sortilin, Column 4: Phalloidin & WGA, Column 5: TOM20 & HSP60) were visualized together to observe regions of colocalization (Fig. 7a, Supplementary Fig. 7). Histograms of the count of pixels in bin widths of 12.5 pixels (108 nm/pixel, bin width 1.354 µm) were plotted for each marker in a single BM USC using VR headset and Genuage (Fig. 7b) to examine the difference in the spatial distribution of the markers within a cell. In VR-based explorations, TOM20, HSP60, ATF6, GOLPH4, and Beta Tubulin demonstrated non-overlapping regions of the cytosol and overlapping hotspots, yielding spatially variant and distinct histogram shape following organelle interactions and individual marker expressions (Fig. 7b). On the other hand, DAPI, Nucleolin, Sortilin, Concanavalin A, and Phalloidin exhibited more spatially continuous regional expression in the VR-based histograms. Thereby, as illustrated in the VR-based organelle visualizations and interactive quantifications, the incorporation of virtual reality into spatial omics datasets will enable the interactive discovery of spatially modulated patterns in multiplexed molecular datasets from health and disease settings.

## Discussion

Image analysis technology for investigating cell features in biological research has advanced significantly over the past several decades. Finding protein associations within cells through spatial image analysis has the potential to deepen our understanding of their microenvironment and how their structural organization changes as cells transition from normal to abnormal cell behaviors. This work focused on observing spatial organelle distribution in two types of MSCs–BM and UC—and studying their differences. RapMIF was performed using antibodies that target the organelles to understand colocalization across the proteins. Multiplex analysis of organelle markers indicated differences in the spatial organization of different organelle proteins in BM and UC MSCs. Interactions between the proteins were compared using Pearson’s correlation coefficients and colocalization, which revealed differences between BM and UC cells. Pixel-level clustering, morphology, and texture analysis methods also showed a considerable organelle networking difference between the two cell types. Organelle phenotypes and their energy changes have been linked to stem cell fate, signaling, and therefore function^39,40^. For example, the ER-mitochondria linkage has been implicated in lipid and energy metabolism as well as apoptosis signaling^41^. Since organelle phenotypes indicate changes in cell energy that affect cell function, it is important to study these phenotypes to improve future therapeutics.

This study demonstrated a RapMIF protocol to perform spatial profiling of organelles in single cells using 10 protein markers. The targets of these proteins were limited to key organelles, namely mitochondria, the Golgi, ER, the nucleus, and the nucleolus. To obtain a comprehensive understanding of cell behavior, proteins targeting other organelles, such as the peroxisomes, need to be included in the study. An important point that needs to be considered while selecting these proteins is the target organelle. While some proteins colocalize in a single organelle, some are chaperones and therefore move between different cellular components. These proteins can be useful to understand communication between organelles, providing insights into the functionality of the cells. However, it is important to consider multiple roles in the spatial and temporal coordination of such proteins to answer questions about the structural organization of the organelles.

A limitation of this study is the number of cells considered for analysis. Imaging of the sample performed manually at high magnification (60×) is time-consuming, and therefore the number of regions captured was limited. Discarding cells that had image quality issues resulted in a small dataset of 14 cells (7 BM and 7 UC MSCs). Automated imaging is a potential way to overcome this limitation, as it can assist with capturing more regions, and therefore more cells. This study considered two-dimensional (2D) data, but using three-dimensional (3D) high-resolution data could improve organelle network mapping and provide more insights into interactions and colocalization. Additionally, organelle contacts and interdependency between the locations of different organelles could be better understood by using 3D data.

The differences between BM and UC MSCs have been well characterized in terms of regenerative, proliferative, differentiation, and clinical outcomes^18,42–45^. Our study examines the organelle-specific activity differences among single cells from each tissue source. The detected activity differences can be attributed to energetic demand differences from their in situ origin. For example, since UC MSCs are typically derived from infants and BM MSCs originate from older, more mature adults, the difference in each population’s energy needs can be observed in the MSC population. UC MSCs were observed to exhibit higher organelle colocalization patterns (Fig. 3a,b), more variable intensity and expression areas (Fig. 3c,d), and less single-cell variability (Fig. 4d) than BM MSCs, suggesting that UC MSCs may be more uniformly and energetically active to satisfy the high energy demands of rapidly changing infant developmental processes and growth. On the other hand, BM MSCs should be reflective of older, sedentary, less active adults. Thus, we observed more uniform, lower intensity, and lower area expression patterns than UC MSCs (Fig. 3). The similar patterns between BM and UC MSCs verified with superpixel segmentation suggest that these differences are less obvious to the eye and more minutely varying in multiplexed organelle maps (Fig. 6).

Previous studies have investigated energetic differences between BM and UC MSCs^46,47^. For example, by measuring lactate from glycolysis, UC MSCs have been shown to produce less lactate than BM MSCs in hypoxic, normoxic, and hyperoxic conditions. Additionally, UC MSCs can adapt more than BM MSCs to a wider range of oxygen conditions as supported by oxygen consumption rates^46^. UC MSCs were able to alter energetic and metabolic levels in response to oxygen-varying environments compared to other types of MSCs. Our study supports these findings at the single-cell level by illustrating more colocalized organelle patterns that possess more variable intensity over larger spatial areas (Fig. 3). UC MSCs also show less cell-to-cell variability in organelle expression (Fig. 4d). Overall, our findings support the energetically higher and adaptive nature of UC MSCs than BM MSCs that are attributable to energetic demands between the tissue sources of younger and older patients.

While these results could answer some questions about spatial differences, further experiments are needed to answer specific questions about energy. Energy levels between UC MSCs and BM MSCs can be compared by studying how organelles change when cultured in different oxygen conditions^10^. Metabolic enzyme proteins can also be used to study energy differences. Combining live imaging with multiplexing will provide spatial and temporal resolution, thus enabling further understanding of organelle interactions^48^. Comparing the difference in organelle enrichment, such as between mitochondria to cytoplasm ratio and ER abundance, provides another interesting point of research for future inquiry. These differences could also reveal details about the functioning of stem cells before their use in therapies.

Another experimental consideration is phase separation, a spatiotemporal process in cells^49^ and a mechanism used in biomolecular assembly. Various membrane-less organelles are formed through liquid–liquid phase separation; these organelles can be added to the multiplex profiling panel^50^. Combining spatial organelle network analysis with studying phase separation will shed light on the biophysics of the biomolecular condensates^51^. Dynamic regulation of phase separation can also be used to understand how organelle interactions are affected during phase transitions in cellular molecules^52^.

To evaluate the mechanisms of how different protein/organelle phenotypes contribute to cell-specific functions, the transcriptome and proteome levels of primary cells and cell lines could be compared. Cell line experiments are a well-established approach because they are readily available and cost-effective. However, culturing cells in the media makes them lack tissue architecture and heterogeneity. Cells in culture can have different molecular phenotypes from cells in vivo^53^. Therefore, primary MSC cells and MSC cell lines can serve as models to compare cell-specific functions by quantifying the genetic and proteomic differences. Furthermore, the functions of primary cells and cell lines could be examined by designing a staining panel of organelle markers (ATF6, TOM20, β-Tubulin, GOLPH4, HSP60, Nucleolin, and Sortilin) and protein markers indicating proliferation or apoptosis (Ki67, BIM, and Cyclin E). Also, to relate gene expression with the change at the proteome level, RNA targets could be included in the staining panel. With the multiplexed experiments, the proteomic and transcriptomic phenotyping of cell lines and primary cells could provide a complete relationship between phenotypic differences and cellular function.

An accurate, reproducible, multiplexed protein imaging and analysis method could aid in better visualizing and understanding various disease states and microenvironments in single cells, which could then be used to find or design drugs and therapy methods that work best depending on patient needs^54^. Subcellular organelle analysis using proteomics is a useful method for obtaining spatial cellular maps that can reveal details about the structure and functionality of organelles and the cell. The complex spatial organelle interaction is yet to be fully understood in the context of organelle-related diseases^55,56^ such as cancer^57^, aging, and regenerative medicine^58^ to assess their potential in precision medicine and therapeutics.

## Methods

### Cells

Bone marrow‐derived MSCs (BM MSCs) and umbilical cord-derived MSCs (UC MSCs) were obtained from RoosterBio, Inc. The culture media was prepared using 89% ɑ-MEM media (Cat # 12561-049) with L-glutamine, 10% heat-inactivated fetal bovine serum (HI-FBS), and 1% penicillin–streptomycin (Cat # P4333). The culture media was mixed and filtered before use. BM and UC MSCs were cultured in T-75 flasks with 10 mL of culture media. Cell passages were performed when cells reached 75% confluency using Trypsin LE cell detachment media (Cat # 12605-010) at 37 °C. The cells were resuspended in respective culture media after centrifugation at 280 g for 6 min and then seeded on collagen-coated glass coverslips. The cells were then cultured on coverslips for 24 h before fixation. Cells were then fixed in 1.6% paraformaldehyde in PBS for 10 min at room temperature, followed by another PBS washing and multiplexed staining of organelle protein markers. Duplicate experiments were used for each measurement. Cells were thawed at passage 2 and cultured until passage 20. BM and UC cells were from similar passage numbers.

### Antibodies

The primary antibodies considered for this study were ATF6 (ab263955, Abcam), β-tubulin (sc-5274, Santa Cruz Biotechnology), GOLPH4/GPP130 (ab197595, Abcam), HSP60 (ab224528, Abcam), Nucleolin (ab226113, Abcam), Sortilin (ab263873, Abcam), Tom20 (sc-17764, Santa Cruz Biotechnology), Phalloidin (A34055, Invitrogen), Wheat Germ Agglutinin (W32466, Invitrogen), and Concanavalin A (C11252, Invitrogen). They were used for cell segmentation and additional organelle markers (Supplementary Table 1). The animal source and dilution of unconjugated antibodies used are as follows: ATF6 (rabbit, 1:200), β-tubulin (mouse, 1:200), GOLPH4 (rabbit, 1:500), HSP60 (rabbit, 1:200), Nucleolin (rabbit, 1:250), Sortilin (rabbit, 1:100) and Tom20 (mouse, 1:200). The dilutions of the antibodies were optimized by performing multiple rounds of IF assays to improve reproducibility.

### Antibody conjugation

The primary antibodies were conjugated with fluorescent dyes using a rapid conjugation kit (ab269823, Abcam). For each 10µL of primary antibody, 1µL of modifier reagent was added and mixed gently. The lyophilized powder was dissolved in 10µL PBS (D8537, Sigma-Aldrich), and 1µL of this solution was added to each antibody and mixed gently. The mixture was incubated at room temperature for 15 min in the dark. After incubation, 1µL of quencher reagent was added for each 10µL of antibody used and was then gently mixed. In this experiment, the antibodies were conjugated to Alexa Fluor 647, which is a bright dye with less background fluorescence^25^. While Alexa Fluor 488 is the brightest among the dyes, the channel has higher background fluorescence. Alexa Fluor 555 is the weakest dye, suitable for staining proteins with high abundance and high affinity.

### Rapid multiplexed immunofluorescence

BM-MSCs and UC-MSCs were stained with 10 markers using a total of 8 cycles (Supplementary Table 2). The cells were permeabilized using 0.5% Triton X-100 for 10 min at room temperature and washed three times with PBS. At the start of each cycle, blocking was performed with Cell Staining Media (CSM containing 0.5% BSA, 0.02% sodium azide in PBS), 0.5% BSA, and 0.02% Sodium Azide (contains PBS 1x) for 1 h at RT. After blocking, the coverslip with the cells was incubated at RT for 1 h with diluted primary or conjugated antibody (250–500µL per coverslip), followed by four washes with 1 × PBS for 5 min. All primary antibodies (conjugated and unconjugated) were diluted in CSM. Since the experiment consisted of both conjugated and unconjugated antibodies, indirect immunofluorescence was performed in the first cycle using the unconjugated antibodies. For indirect immunofluorescence, after incubating with the primary antibodies, the coverslip was incubated in secondary antibodies diluted in PBS for 1 h at RT, followed by four washes with 1 × PBS for 5 min. The coverslip was then incubated with DAPI (D1306, Invitrogen) diluted in PBS for 10 min at RT, followed by four washes using 1 × PBS for 5 min each. 1 × PBS was used as the imaging buffer on the coverslip. After imaging, bleaching was performed using a freshly prepared bleaching buffer consisting of 4.5% (wt/vol) H_2_O_2_ and 20 mM NaOH in PBS for 1 h at RT with white light. The sample was imaged to ensure that the fluorescence signal had fallen to background levels (Supplementary Figs. 8 and 9). The coverslip was washed with 1 × PBS three times before starting the next cycle.

### Imaging

A wide-field microscope, Nikon Eclipse TE2000-U, was used for fluorescence imaging. For each cycle of imaging, 2–3 channels were captured: Channel 1 detects DAPI/Hoechst at 360 nm, while Channels 2–4 detect fluorophores at Alexa Fluor 488 nm (FITC), 555 nm (TRITC), and 647 nm (Cy5), respectively. The exposure time varied between the markers and the cells. The sample was imaged with a 60X oil lens, resulting in a high-resolution acquisition at 0.1083 µm/pixel. Each imaging region was imaged across 16 z-stacks with 0.5 µm/stack. The best focus z slices of each image were used for analysis. The images were stored as 16-bit multichannel images in ND2 format. They were converted to TIFF format using ImageJ and saved as single or multi-channel grayscale images. Each marker was thresholded with the Otsu method for all analyses^59^.

### Quality control

Registration was performed on sequential images from multiple cycles of immunofluorescence to adjust differences in alignment using the RNAscope HiPlex Image Registration Software^60^. DAPI channel images of each cycle were aligned using the DAPI channel image of the first cycle as a reference to obtain the registration transforms, which were then applied to the remaining channels of each cycle. After registration, the best cells were manually selected through visual inspection based on image quality. For example, unreliable regions with intensity values too close to the local background (insufficient contrast) or saturated intensity values (making it hard to distinguish features due to high intensity) were filtered out. Cells that had overlapping cytosol and cells which were touching the image borders were also discarded. This quality control step resulted in 7 BM MSCs and 7 UC MSCs for analysis. Background subtraction was performed using a rolling ball algorithm in ImageJ to remove the background signal from the images. Finally, the pixel intensities were thresholded by calculating the lower 20 percentile (background pixels) and the upper 99.9 percentile (saturated pixels), rescaling the intensities using the two percentiles between 0 and 1 and clipping the values that were lower than 0 and greater than 1. The cells were segmented in ImageJ using cell masks obtained from the Phalloidin marker outline (Supplementary Fig. 10).

### Scatterplot

Scatter plots of intensity were obtained by random sampling of 50,000 pixels for each marker pair colocalized in the same organelle in BM-MSCs and UC-MSCs. The intensity values were min–max normalized. The plots were colored using kernel density estimates obtained from Gaussian kernels.

### Pearson’s correlation

Pearson’s correlation coefficient was calculated for each cell between the markers to evaluate the extent of colocalization between the organelle proteins in the cell. The results were averaged across the cells, and the variance of the values was calculated. The average and variance of correlation coefficient values were visualized as a heatmap with dendrograms for BM and UC cells. A clustered heatmap of Pearson's correlation was displayed with each pair of markers on the x-axis and each cell type on the y-axis. This result is useful to compare the organelle colocalization between BM and UC cells.

### Pixel overlap colocalization

The pixel overlap colocalization between two given markers was calculated by counting the number of nonzero pixels that occupy the same coordinate. This value was normalized by dividing by the total area of both markers to obtain a final fraction of the common area covered by two markers.

### Statistical analysis

The Kolmogorov–Smirnov (KS) hypothesis test was conducted between organelle marker pairs targeting the Golgi (GOLPH4 and Sortilin) and mitochondria (TOM20 and HSP60). For each cell, the center of mass was computed from the segmented mask. Then, each marker’s pixel distance to the center of mass was calculated to convert 2D spatial image coordinates to 1D spatial distributions (Supplementary Fig. 11). To determine if these spatial distributions differed from one another, the KS test is performed on the 1D spatial distribution (histogram). Since cell morphology is roughly symmetrical, the test examines differences in organelle spatial expression patterns between the nucleus and membrane. The null hypothesis considered was that similar proteins express similar spatial distribution within each cell.

### Pairwise analysis

For each marker, multiple, Otsu-thresholded single-cell images were selected and the intensity values were converted into a column. The intensity values were min–max normalized. Pearson’s correlation was calculated using intensity values of all marker pairs for each cell in the marker pair and the result was plotted as boxplots. Similarly, to verify Pearson values, pixel overlap colocalization between the markers was also calculated and the values were plotted as boxplots.

### Cell area and intensity

For each marker, multiple single-cell images were selected and the average intensity values per cell were plotted as boxplots. Similarly, the area of each marker per cell was calculated by summing the number of non-zero pixels after morphological opening and closing. The area values were also plotted as boxplots.

### Pixel clustering

Pixel phenotypes were clustered in a two-step clustering pipeline. From each pixel location within the cell-segmented region, the intensity value of each marker expression was extracted. The resulting feature matrix consisted of n rows of a total number of pixels and p columns of marker expression. Each column of the feature matrix was min–max normalized. To determine the optimal number of clusters, the elbow method was implemented on the cluster scores for various numbers of clusters. The elbow point was determined to be 10. Thus, the K-Means clustering algorithm was used to cluster the pixels of the 5 markers into 10 clusters. The resultant clusters were then uniquely colored on the original cellular image. To look for any organelle pattern in the clusters, the K-Means clustering results were compared with images of markers grouped according to their organelle affinity and multiplied together pixel-wise.

### Super-pixel segmentation and texture analysis

The images were segmented into superpixels using scikit-image^61^ (a Python library for scientific image processing) and K-Means clustering into superpixels^37^. Texture features were calculated on the superpixels: pixel intensity, energy Laplacian, modified Laplacian, diagonal Laplacian, variance Laplacian, and gray level variance. Pixel intensity computes the mean of the intensities within each superpixel. Energy Laplacian computes the square of the Laplacian of the image from skimage^61^. Modified Laplacian also uses a Laplace function but with two kernels in which one is the transpose of the other; then, the resulting images are summed. Diagonal Laplacian uses convolution with 4 kernels to produce the resulting images that are then summed. Variance Laplacian consists of the square of the difference between the image Laplace and the mean of the image Laplace. Gray level variance involves the square of the difference between the image and the mean of the image. The Python code that contains all the texture functions is labeled “texture_analysis_functions.py” and is available on the Github. The superpixels and texture features were plotted as images and heatmaps were obtained for each marker to compare BM and UC MSCs.

### Virtual reality visualization

The images of organelle markers in BM and UC cells stored as TIF files were converted into Neuroimaging Informatics Technology Initiative file format (NIfTI or .nii) using ImageJ^38^. These files were opened in ConfocalVR software to visualize them in an immersive 3D format^62^. The pixel data was also visualized in another VR software, Genuage, and histograms of the pixel counts were obtained using histogram bins drawn using controllers^63^.

### CellProfiler

Morphological features, such as area, major axis, and minor axis, were obtained for each cell using a CellProfiler pipeline^64^, and were then plotted as heatmaps with hierarchical clustering. To first identify and segment the nuclei of cells, “IdentifyPrimaryObject” was used on the DAPI marker with the global two-class Otsu thresholding method. Using the segmented nuclei, the “IdentifySecondaryObject” module was then used to segment the general shapes of the organelles marked by each marker using the same thresholding method. After images of every marker were segmented, all the size/shape features were measured using the “MeasureObjectSizeShape'' module for each of the markers and exported into an excel file. The data stored in an excel spreadsheet was manually sorted to extract and analyze only the features of interest, which were the area, major axis, and minor axis of each marker. The measurements of interest were converted into CSV files for visualization. Heatmaps were generated using Python with dendrograms to compare the biophysical measurements and to determine any close relationships or associations between markers and between the two cell types (UC and BM). The z-score was calculated to normalize the output data.

**Table:** 

Software and algorithms	
Cellprofiler	https://cellprofiler.org/
Numpy	https://numpy.org/
Scipy	https://scipy.org/
Dask	https://ml.dask.org/clustering.html
Statsannotations	https://github.com/trevismd/statannotations
Pandas	https://github.com/pandas-dev/pandas
Seaborn	https://github.com/mwaskom/seaborn
Code related to this study	https://github.com/coskunlab/Spatial-organelle-networks

## Supplementary Information

Supplementary Information.

## Data Availability

Relevant data and analysis results are available at https://doi.org/10.5281/zenodo.6468563 and https://github.com/coskunlab/Spatial-organelle-networks.

## Code availability

The codes are available at https://github.com/coskunlab/Spatial-organelle-networks.
