# Supplementary material for: Spatial subcellular organelle networks in single cells
Source: Sci Rep. 2023 Apr 1;13:5374. doi: 10.1038/s41598-023-32474-y (PMC10067843; doi:10.1038/s41598-023-32474-y)
Supplement: Supplementary file 1 — Supplementary Information. [file 41598_2023_32474_MOESM1_ESM.pdf]

Spatial subcellular organelle networks in single cells

Mythreye Venkatesan[1,2,3*], Nicholas Zhang[1,2*], Benoit Marteau[3], Yukina Yajima[1], Nerea Ortiz De Zarate Garcia[1,4], Zhou Fang[1], Thomas Hu[1,3], Shuangyi Cai[1], Adam Ford[1], Harrison Olszewski[1], Andrew Borst[1], and Ahmet F. Coskun[1,2,5#]

[1] Wallace H. Coulter Department of Biomedical Engineering, Georgia Institute of Technology and Emory University, Atlanta, GA, USA

[2] Interdisciplinary Bioengineering Graduate Program, Georgia Institute of Technology, Atlanta, GA, USA

[3] School of Electrical and Computer Engineering, Georgia Institute of Technology, Atlanta, GA, USA

[4] Departamento de Bioingenieria e Ingenieria Aeroespacial, Universidad Carlos III de Madrid

[5] Parker H. Petit Institute for Bioengineering and Bioscience, Georgia Institute of Technology, Atlanta, GA, USA

*Equal contribution

[#]Corresponding author

Ahmet F. Coskun, Ph.D. (ahmet.coskun@bme.gatech.edu)

a

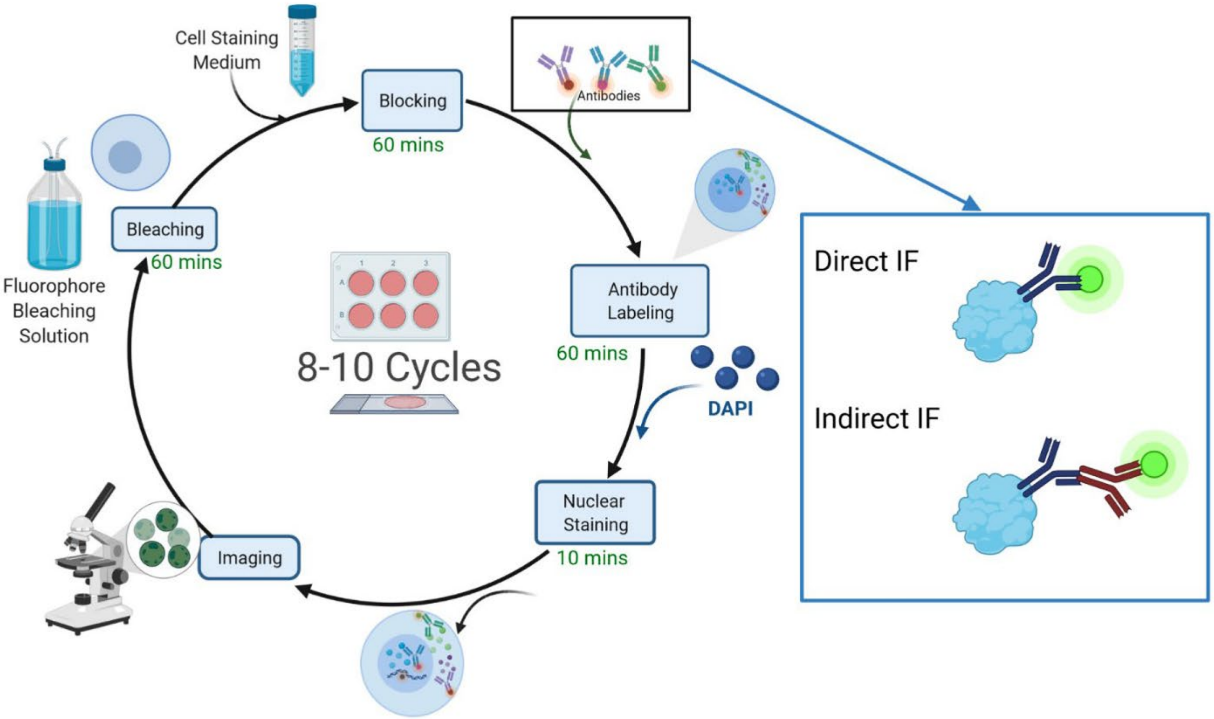

Cell Staining Medium
Blocking
60 mins
Antibodies
Antibody Labeling
60 mins
DAPI
Nuclear Staining
10 mins
Imaging
Bleaching
60 mins
Fluorophore Bleaching Solution
8-10 Cycles
Direct IF
Indirect IF

b

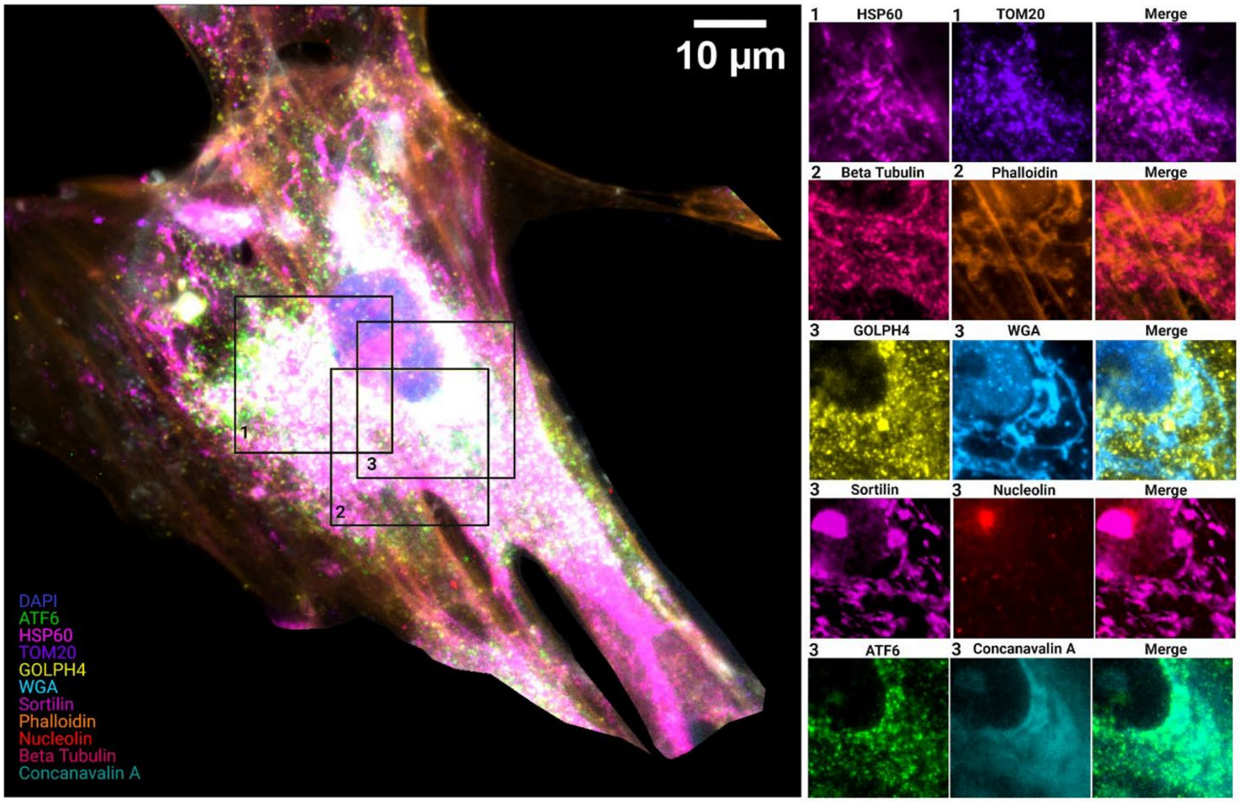

10 µm
DAPI
ATF6
HSP60
TOM20
GOLPH4
WGA
Sortilin
Phalloidin
Nucleolin
Beta Tubulin
Concanavalin A
1 HSP60
1 TOM20
Merge
2 Beta Tubulin
2 Phalloidin
Merge
3 GOLPH4
3 WGA
Merge
3 Sortilin
3 Nucleolin
Merge
3 ATF6
3 Concanavalin A
Merge

Supplementary Figure 1. Rapid multiplexed immunofluorescence for high-dimensional spatial profiling in single cells.

(a) Timeline for rapid multiplexed immunofluorescence staining. Each cycle consists of up to 3 antibodies plus DAPI. In between subsequent cycles, the antibodies were bleached and the same sample was re-blocked and stained with a new set of antibodies. Inset shows the difference between direct and indirect IF. While direct staining is a single-step process, indirect staining is a two-step requiring a primary antibody and a secondary antibody and is, therefore, more time-consuming. The duration of each step is labeled in green. Created with BioRender.com.

(b) 10-plex visualization of organelle markers in BM MSCs. On the right, zoomed-in regions are shown to illustrate regions of organelle colocalization and overlap of organelle marker presence. Row 1: HSP60 & TOM20, Row 2: Beta Tubulin & Phalloidin, Row 3: GOLPH4 & WGA, Row 4: Sortilin & Nucleolin, Row 5: ATF6 & Concanavalin A. The third column indicates the merge of the two markers in the first two columns.

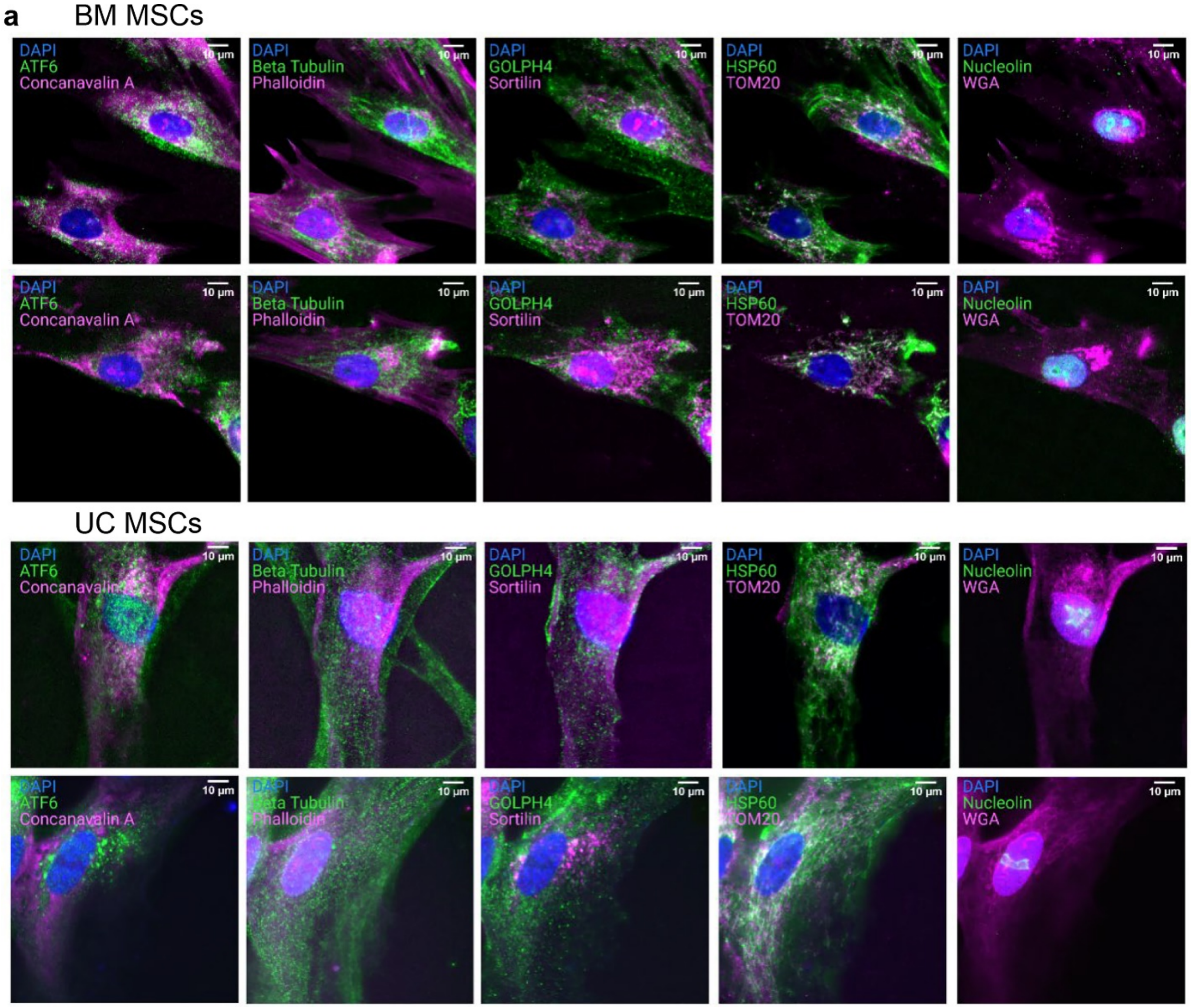

a
BM MSCs
UC MSCs
DAPI
ATF6
Concanavalin A
DAPI
Beta Tubulin
Phalloidin
DAPI
GOLPH4
Sortilin
DAPI
HSP60
TOM20
DAPI
Nucleolin
WGA
10 µm

Supplementary Figure 2. 10-plex Organelle mapping in mesenchymal stem cells.

(a) Visualization of organelle markers in single cells from BM MSCs. Each row corresponds to a unique cell and each image displays two organelle markers. Column 1: ATF6 (green) & Concanavalin A (magenta), Column 2: Beta Tubulin (green) & Phalloidin (magenta), Column 3: GOLPH4 (green) & Sortilin (magenta), Column 4: HSP60 (green) & TOM20 (magenta), Column 5: Nucleolin (green) & WGA (magenta). All scale bars 10 μm.

(b) Visualization of organelle markers in single cells from UC MSCs. Each row corresponds to a unique cell and each image displays two organelle markers. Column 1: ATF6 (green) & Concanavalin A (magenta), Column 2: Beta Tubulin (green) & Phalloidin (magenta), Column 3: GOLPH4 (green) & Sortilin (magenta), Column 4: HSP60 (green) & TOM20 (magenta), Column 5: Nucleolin (green) & WGA (magenta). All scale bars 10 μm.

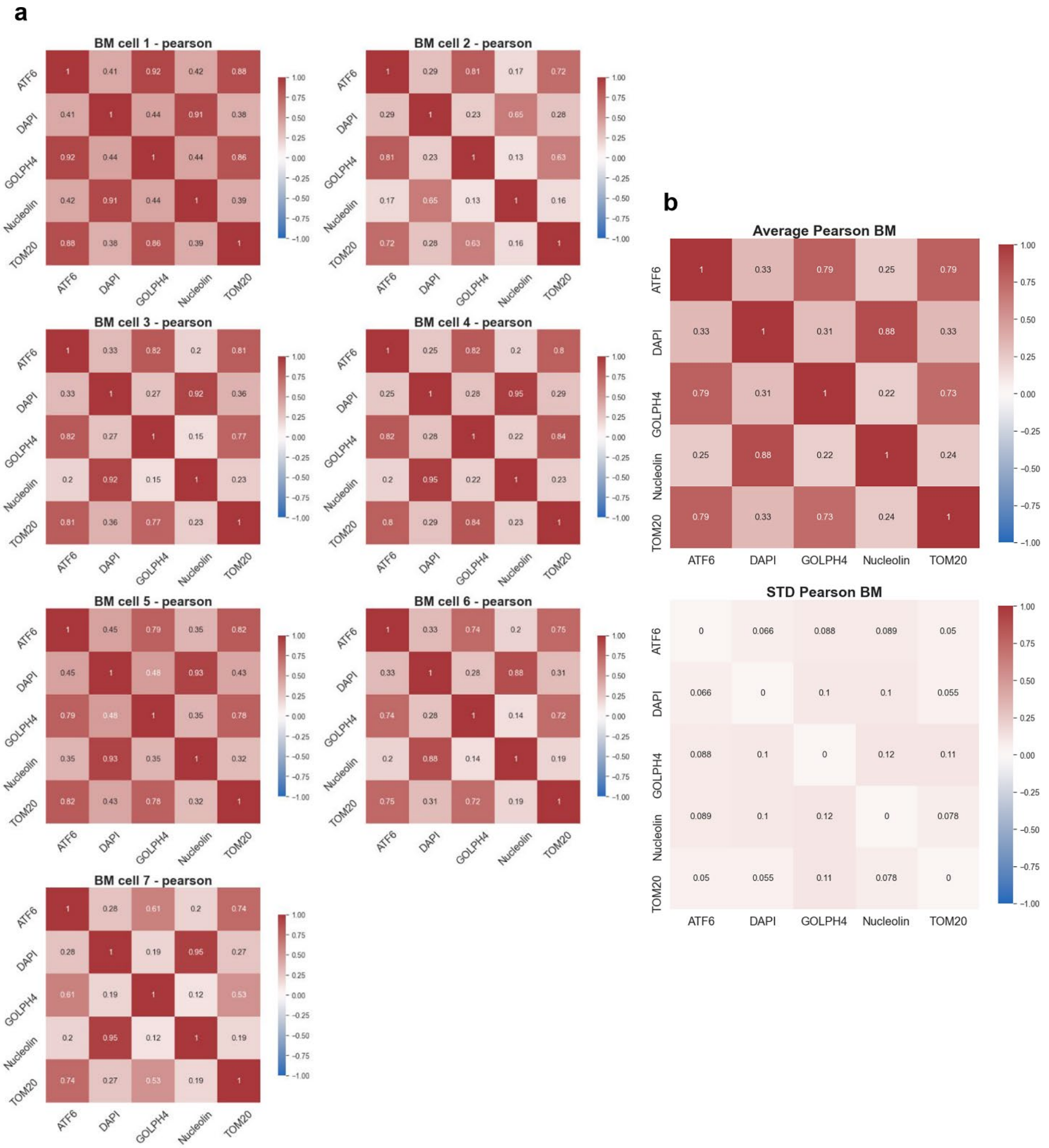
a
BM cell 1 - pearson
1 0.41 0.92 0.42 0.88
0.41 1 0.44 0.91 0.38
0.92 0.44 1 0.44 0.86
0.42 0.91 0.44 1 0.39
0.88 0.38 0.86 0.39 1
BM cell 2 - pearson
1 0.29 0.81 0.17 0.72
0.29 1 0.23 0.65 0.28
0.81 0.23 1 0.13 0.63
0.17 0.65 0.13 1 0.16
0.72 0.28 0.63 0.16 1
BM cell 3 - pearson
1 0.33 0.82 0.2 0.81
0.33 1 0.27 0.92 0.36
0.82 0.27 1 0.15 0.77
0.2 0.92 0.15 1 0.23
0.81 0.36 0.77 0.23 1
BM cell 4 - pearson
1 0.25 0.82 0.2 0.8
0.25 1 0.28 0.95 0.29
0.82 0.28 1 0.22 0.84
0.2 0.95 0.22 1 0.23
0.8 0.29 0.84 0.23 1
BM cell 5 - pearson
1 0.45 0.79 0.35 0.82
0.45 1 0.48 0.93 0.43
0.79 0.48 1 0.35 0.78
0.35 0.93 0.35 1 0.32
0.82 0.43 0.78 0.32 1
BM cell 6 - pearson
1 0.33 0.74 0.2 0.75
0.33 1 0.28 0.88 0.31
0.74 0.28 1 0.14 0.72
0.2 0.88 0.14 1 0.19
0.75 0.31 0.72 0.19 1
BM cell 7 - pearson
1 0.28 0.61 0.2 0.74
0.28 1 0.19 0.95 0.27
0.61 0.19 1 0.12 0.53
0.2 0.95 0.12 1 0.19
0.74 0.27 0.53 0.19 1
ATF6
DAPI
GOLPH4
Nucleolin
TOM20
1.00
0.75
0.50
0.25
0.00
−0.25
−0.50
−0.75
−1.00
b
Average Pearson BM
1 0.33 0.79 0.25 0.79
0.33 1 0.31 0.88 0.33
0.79 0.31 1 0.22 0.73
0.25 0.88 0.22 1 0.24
0.79 0.33 0.73 0.24 1
STD Pearson BM
0 0.066 0.088 0.089 0.05
0.066 0 0.1 0.1 0.055
0.088 0.1 0 0.12 0.11
0.089 0.1 0.12 0 0.078
0.05 0.055 0.11 0.078 0

Supplementary Figure 3. Pearson's correlation of markers in BM MSCs (n=7).

(a) Correlation values of the total intensity of 5 markers (ATF6, DAPI, GOLPH4, Nucleolin, TOM20) in BM MSCs were calculated per marker for each cell using Pearson's Correlation and plotted as heatmaps. Pearson's correlation values range from -1 (blue) to +1 (red).

(b) Average (top) and standard deviation (bottom) of Pearson's correlation of intensity per marker across all BM MSCs for 5 markers (ATF6, DAPI, GOLPH4, Nucleolin, TOM20). The color bar ranges from -1 (blue) to +1 (red). Standard deviation values are close to 0 while average values agree well with every single cell in (a), indicating a more uniform organelle pattern among BM MSCs.

a

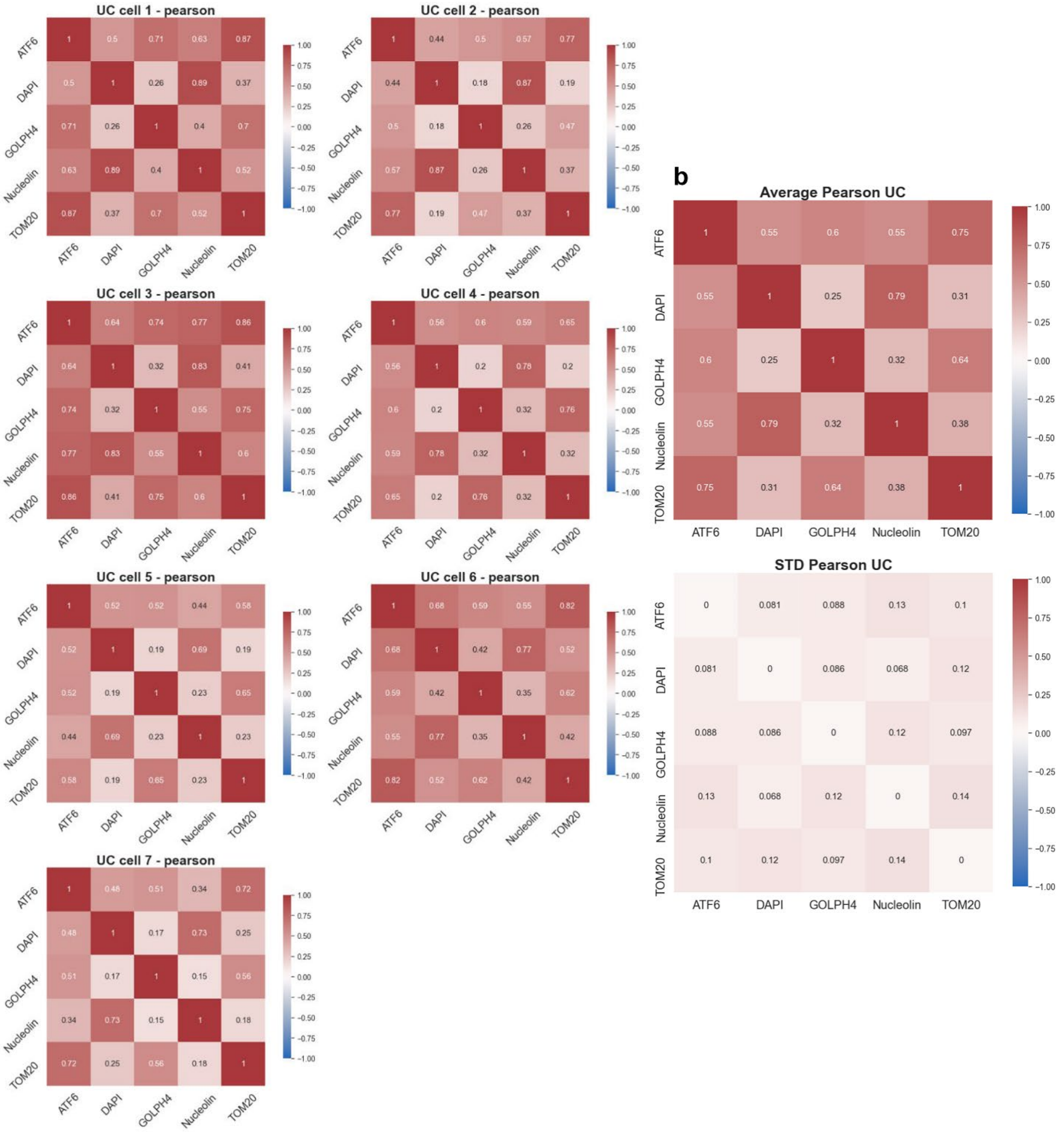
UC cell 1 - pearson
UC cell 2 - pearson
UC cell 3 - pearson
UC cell 4 - pearson
UC cell 5 - pearson
UC cell 6 - pearson
UC cell 7 - pearson
ATF6
DAPI
GOLPH4
Nucleolin
TOM20
1.00
0.75
0.50
0.25
0.00
−0.25
−0.50
−0.75
−1.00
b
Average Pearson UC
ATF6: 1, 0.55, 0.6, 0.55, 0.75
DAPI: 0.55, 1, 0.25, 0.79, 0.31
GOLPH4: 0.6, 0.25, 1, 0.32, 0.64
Nucleolin: 0.55, 0.79, 0.32, 1, 0.38
TOM20: 0.75, 0.31, 0.64, 0.38, 1
STD Pearson UC
ATF6: 0, 0.081, 0.088, 0.13, 0.1
DAPI: 0.081, 0, 0.086, 0.068, 0.12
GOLPH4: 0.088, 0.086, 0, 0.12, 0.097
Nucleolin: 0.13, 0.068, 0.12, 0, 0.14
TOM20: 0.1, 0.12, 0.097, 0.14, 0

Supplementary Figure 4. Pearson's correlation of markers in UC MSCs (n=7).

(a) Correlation values of the total intensity of 5 markers (ATF6, DAPI, GOLPH4, Nucleolin, TOM20) in UC MSCs were calculated per marker for each cell using Pearson's Correlation and plotted as heatmaps. Pearson's correlation values range from -1 (blue) to +1 (red).

(b) Average (top) and standard deviation (bottom) of Pearson's correlation of intensity per marker across all UC MSCs for 5 markers (ATF6, DAPI, GOLPH4, Nucleolin, TOM20). The color bar ranges from -1 (blue) to +1 (red). Standard deviation values are close to 0 while average values agree well with every single cell in (a) except for UC cells 5 and 7, indicating a less uniform organelle pattern among UC MSCs.

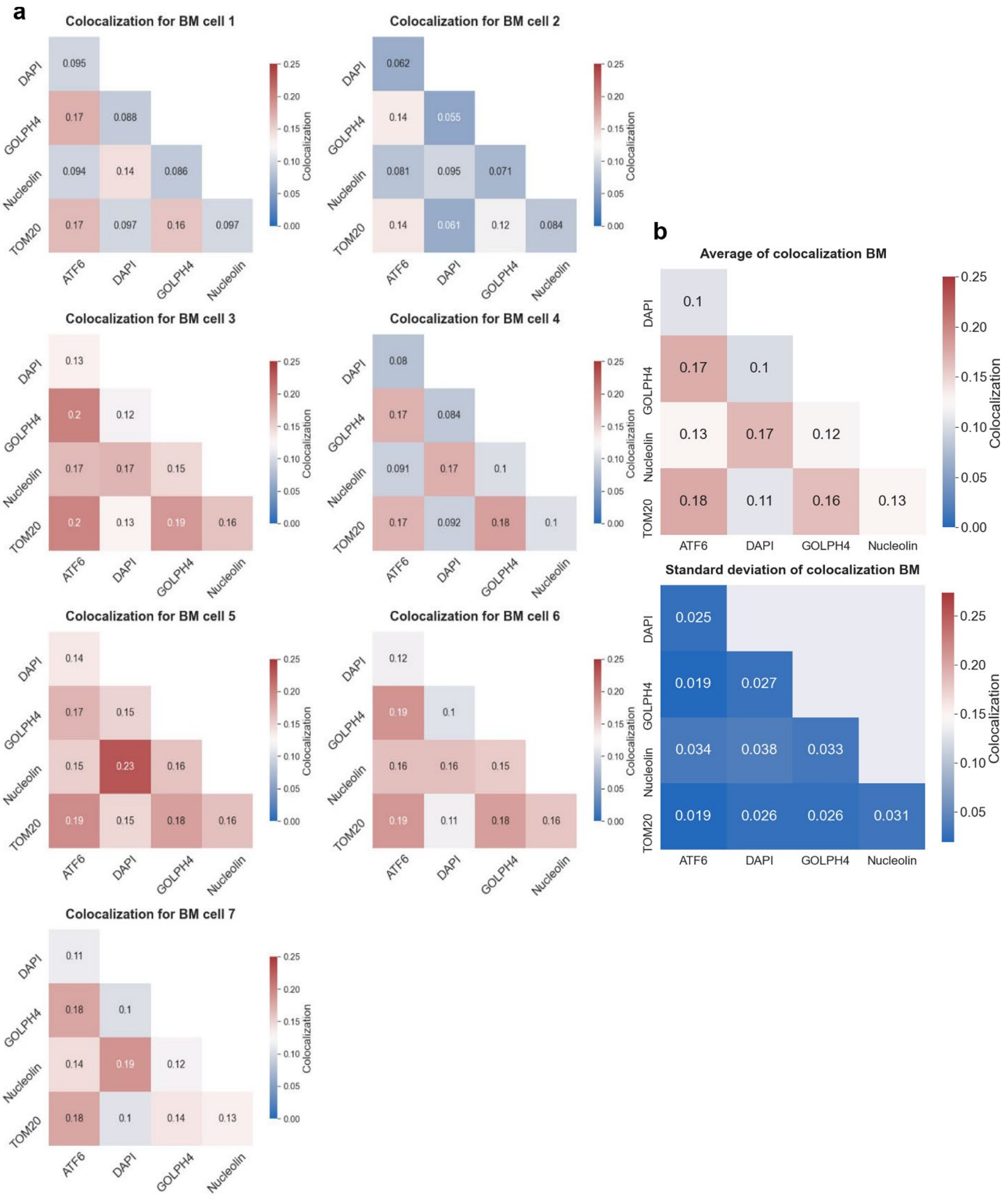
a
Colocalization for BM cell 1
DAPI 0.095
GOLPH4 0.17 0.088
Nucleolin 0.094 0.14 0.086
TOM20 0.17 0.097 0.16 0.097
ATF6 DAPI GOLPH4 Nucleolin
Colocalization
0.00 0.05 0.10 0.15 0.20 0.25
Colocalization for BM cell 2
DAPI 0.062
GOLPH4 0.14 0.055
Nucleolin 0.081 0.095 0.071
TOM20 0.14 0.061 0.12 0.084
ATF6 DAPI GOLPH4 Nucleolin
Colocalization for BM cell 3
DAPI 0.13
GOLPH4 0.2 0.12
Nucleolin 0.17 0.17 0.15
TOM20 0.2 0.13 0.19 0.16
ATF6 DAPI GOLPH4 Nucleolin
Colocalization for BM cell 4
DAPI 0.08
GOLPH4 0.17 0.084
Nucleolin 0.091 0.17 0.1
TOM20 0.17 0.092 0.18 0.1
ATF6 DAPI GOLPH4 Nucleolin
Colocalization for BM cell 5
DAPI 0.14
GOLPH4 0.17 0.15
Nucleolin 0.15 0.23 0.16
TOM20 0.19 0.15 0.18 0.16
ATF6 DAPI GOLPH4 Nucleolin
Colocalization for BM cell 6
DAPI 0.12
GOLPH4 0.19 0.1
Nucleolin 0.16 0.16 0.15
TOM20 0.19 0.11 0.18 0.16
ATF6 DAPI GOLPH4 Nucleolin
Colocalization for BM cell 7
DAPI 0.11
GOLPH4 0.18 0.1
Nucleolin 0.14 0.19 0.12
TOM20 0.18 0.1 0.14 0.13
ATF6 DAPI GOLPH4 Nucleolin
b
Average of colocalization BM
DAPI 0.1
GOLPH4 0.17 0.1
Nucleolin 0.13 0.17 0.12
TOM20 0.18 0.11 0.16 0.13
ATF6 DAPI GOLPH4 Nucleolin
Colocalization
0.00 0.05 0.10 0.15 0.20 0.25
Standard deviation of colocalization BM
DAPI 0.025
GOLPH4 0.019 0.027
Nucleolin 0.034 0.038 0.033
TOM20 0.019 0.026 0.026 0.031
ATF6 DAPI GOLPH4 Nucleolin
Colocalization
0.05 0.10 0.15 0.20 0.25

Supplementary Figure 5. Pixel overlap colocalization of markers in BM MSCs (n=7).

(a) Pixel overlap colocalization of 5 markers (ATF6, DAPI, GOLPH4, Nucleolin, TOM20) in BM MSCs per marker for each cell (n=7) plotted as heatmaps. The color bar ranges from 0 (blue) to 0.25 (red) and indicates the fraction of the cell occupied by the marker pair. BM cells 1, 2, and 4 exhibit slightly different organelle patterns than BM cells 3, 5, 6, and 7. DAPI & Nucleolin, ATF6 & TOM20, ATF6 & GOLPH4, and TOM20 & GOLPH4 exhibit the highest pixel overlap colocalizations.

(b) Average (top) and standard deviation (bottom) of pixel overlap colocalization per marker across all BM MSCs for 5 markers (ATF6, DAPI, GOLPH4, Nucleolin, TOM20). The color bar ranges from 0 (blue) to 0.25 (red). DAPI & Nucleolin, ATF6 & TOM20, ATF6 & GOLPH4, and TOM20 & GOLPH4 exhibit the highest pixel overlap colocalizations. Standard deviations are mostly low, indicating less single-cell variation.

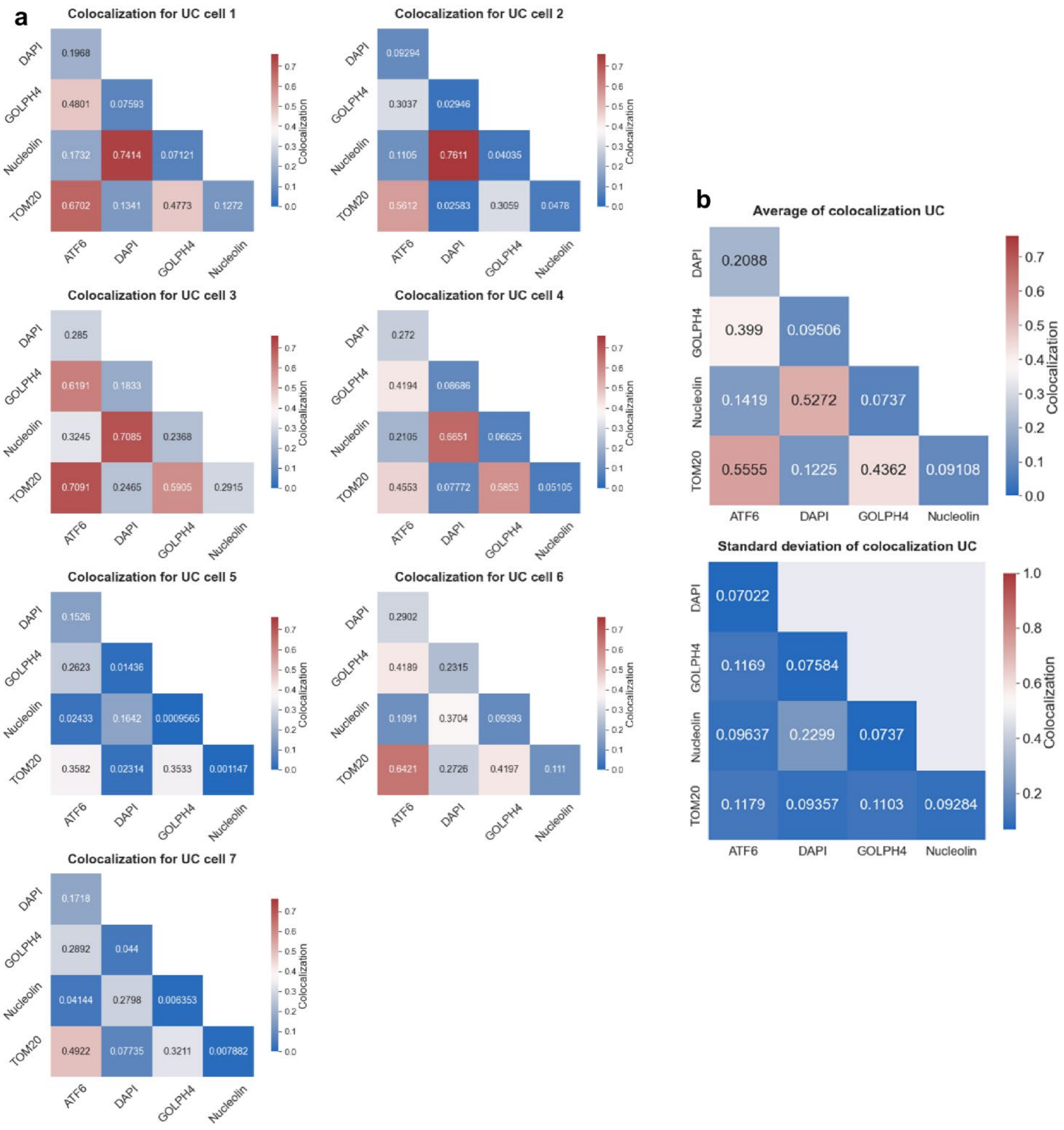
a
Colocalization for UC cell 1
0.1968
0.4801
0.07593
0.1732
0.7414
0.07121
0.6702
0.1341
0.4773
0.1272
Colocalization for UC cell 2
0.09294
0.3037
0.02946
0.1105
0.7611
0.04035
0.5612
0.02583
0.3059
0.0478
Colocalization for UC cell 3
0.285
0.6191
0.1833
0.3245
0.7085
0.2368
0.7091
0.2465
0.5905
0.2915
Colocalization for UC cell 4
0.272
0.4194
0.08686
0.2105
0.6651
0.06625
0.4553
0.07772
0.5853
0.05105
Colocalization for UC cell 5
0.1526
0.2623
0.01436
0.02433
0.1642
0.0009565
0.3582
0.02314
0.3533
0.001147
Colocalization for UC cell 6
0.2902
0.4189
0.2315
0.1091
0.3704
0.09393
0.6421
0.2726
0.4197
0.111
Colocalization for UC cell 7
0.1718
0.2892
0.044
0.04144
0.2798
0.006353
0.4922
0.07735
0.3211
0.007882
DAPI
GOLPH4
Nucleolin
TOM20
ATF6
Colocalization
b
Average of colocalization UC
0.2088
0.399
0.09506
0.1419
0.5272
0.0737
0.5555
0.1225
0.4362
0.09108
Standard deviation of colocalization UC
0.07022
0.1169
0.07584
0.09637
0.2299
0.0737
0.1179
0.09357
0.1103
0.09284

Supplementary Figure 6. Pixel overlap colocalization of markers in UC MSCs (n=7).

(a) Pixel overlap colocalization of 5 markers (ATF6, DAPI, GOLPH4, Nucleolin, TOM20) in UC MSCs per marker for each cell (n=7) plotted as heatmaps. The color bar ranges from 0 (blue) to 0.76 (red). TOM20 and ATF6 show high colocalization in all cells except UC cells 4 and 5, suggesting crosstalk between mitochondria and ER in most UC MSCs. Nucleolin and DAPI also exhibit high colocalization, indicating some segregation of nuclear and cytosolic organelles.

(b) Average (top) and standard deviation (bottom) of pixel overlap colocalization per marker across all BM MSCs for 5 markers (ATF6, DAPI, GOLPH4, Nucleolin, TOM20). The color bar ranges from 0 (blue) to 0.76 (red) for average and 0 (blue) to 1 (red) for standard deviation. The highest average colocalization exists between DAPI & Nucleolin, and ATF6 & TOM20. Thus, many UC MSCs exhibit crosstalk between mitochondria and ER while nuclear organelles are more separated from cytosolic ones. The low standard deviation shows most cells behave similarly.

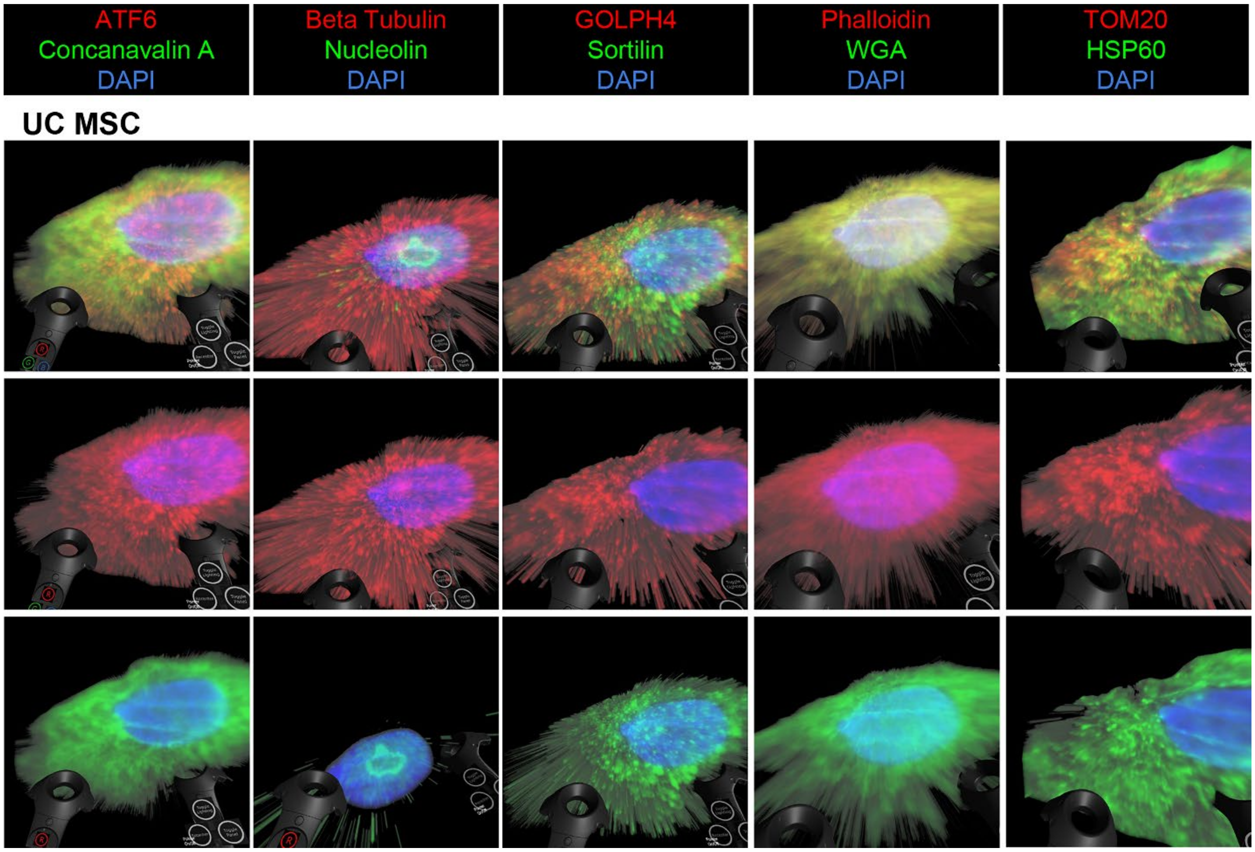
ATF6
Concanavalin A
DAPI
Beta Tubulin
Nucleolin
DAPI
GOLPH4
Sortilin
DAPI
Phalloidin
WGA
DAPI
TOM20
HSP60
DAPI
UC MSC

Supplementary Figure 7. VR-based visualization of organelle imaging data in MSCs.

Visualization of organelle protein images (10 markers) for one UC MSC in ConfocalVR. For each cell, the top shows the RGB image of the combined markers, while the bottom two show a single marker image in either red or green with DAPI. Different organelle markers are illustrated across the columns. Merged images are shown in the first row. Column 1: ATF6 (middle row) & Concanavalin A (bottom row), Column 2: Beta Tubulin (middle row) & Nucleolin (bottom row), Column 3: GOLPH4 (middle row) & Sortilin (bottom row), Column 4: Phalloidin (middle row) & WGA (bottom row), Column 5: TOM20 (middle row) & HSP60 (bottom row). Handset toggle switches used to interact with the image are shown on the left and right sides.

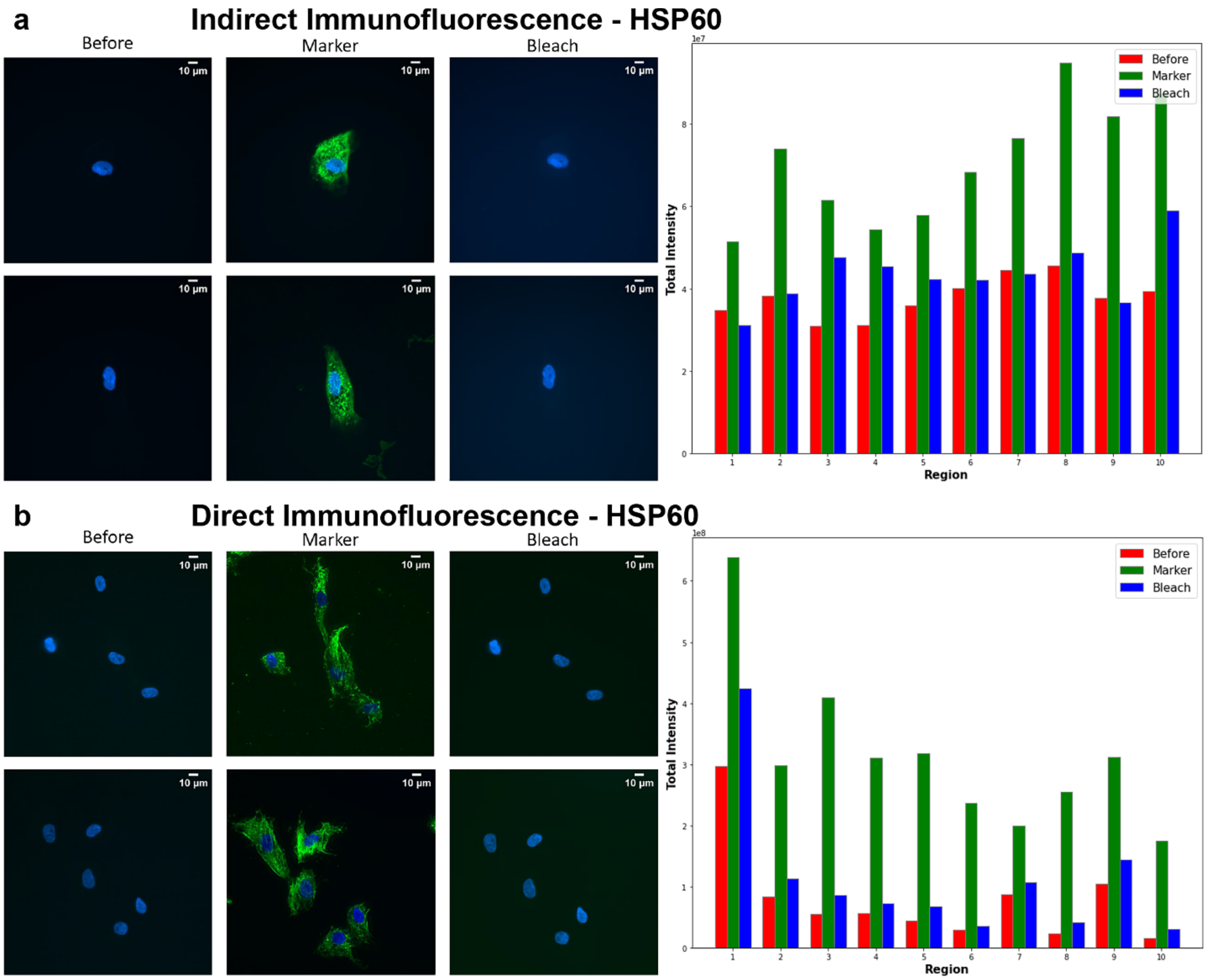
a
Indirect Immunofluorescence - HSP60
Before
Marker
Bleach
10 µm
1e7
Total Intensity
Region
Before
Marker
Bleach
b
Direct Immunofluorescence - HSP60
Before
Marker
Bleach
10 µm
1e8
Total Intensity
Region
Before
Marker
Bleach

Supplementary Figure 8. Validation of bleaching protocol for indirect and direct IF with HSP60.

(a) Images of before staining (left column), staining (middle column), and after bleaching (right column) of HSP60 (green) for indirect IF. Before and after bleaching images confirm signal addition and removal before the next staining cycle. All scale bars 10 µm. On the right, the intensity sum of ten regions before staining, staining, and after bleaching is quantified to validate the effect of the fluorophore bleaching process. Before (red) and after bleach (blue) show comparable intensities while marker (green) illustrates approximately 1.5-2-fold higher intensity.

(b) Images of before staining (left column), staining (middle column), and after bleaching (right column) of HSP60 (green) for direct IF. Before and after bleaching images confirm signal addition and removal before the next staining cycle. Some green background remains after bleaching but this is uniform outside the cell and is easily removed. All scale bars 10 µm. On the right, the intensity sum of ten regions before staining, staining, and after bleaching is quantified to validate the effect of the fluorophore bleaching process. Before (red) and after bleach (blue) show comparable intensities while marker (green) illustrates approximately 2-fold higher intensity.

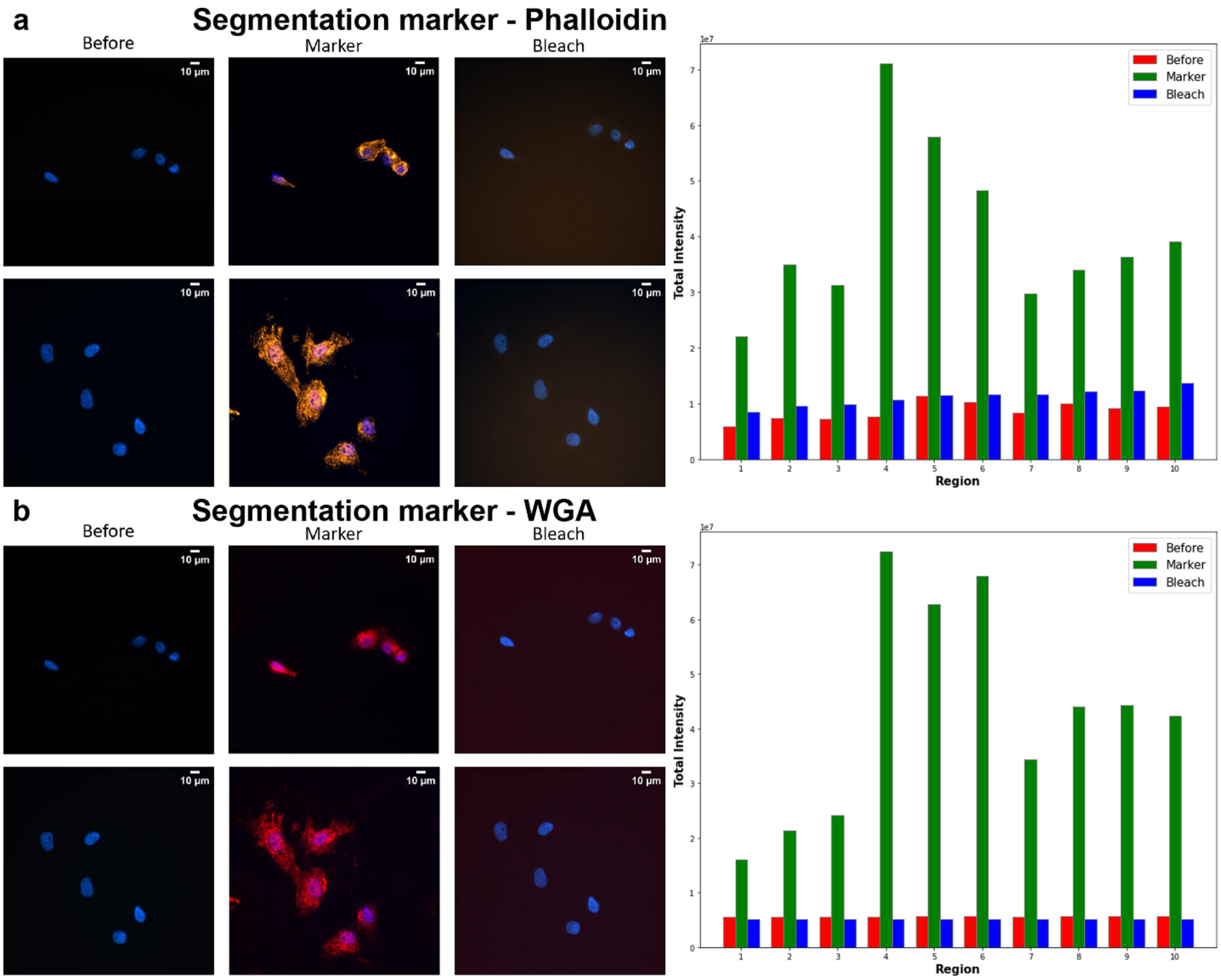
a
Segmentation marker - Phalloidin
Before
Marker
Bleach
10 µm
1e7
Total Intensity
Region
Before
Marker
Bleach
b
Segmentation marker - WGA
Before
Marker
Bleach
10 µm
1e7
Total Intensity
Region
Before
Marker
Bleach

Supplementary Figure 9. Validation of bleaching protocol for segmentation markers Phalloidin and WGA.

(a) Images of before staining (left column), staining (middle column), and after bleaching (right column) of Phalloidin (yellow). Before and after bleaching images confirm signal addition and removal before the next staining cycle. All scale bars 10 µm. On the right, the intensity sum of ten regions before staining, staining, and after bleaching is quantified to validate the effect of the fluorophore bleaching process. Before (red) and after bleach (blue) show comparable intensities while marker (green) illustrates approximately 2-3-fold higher intensity.

(b) Images of before staining (left column), staining (middle column), and after bleaching (right column) of WGA (red). Before and after bleaching images confirm signal addition and removal before the next staining cycle. All scale bars 10 µm. On the right, the intensity sum of ten regions before staining, staining, and after bleaching is quantified to validate the effect of the fluorophore bleaching process. Before (red) and after bleach (blue) show comparable intensities while marker (green) illustrates approximately 2-7-fold higher intensity.

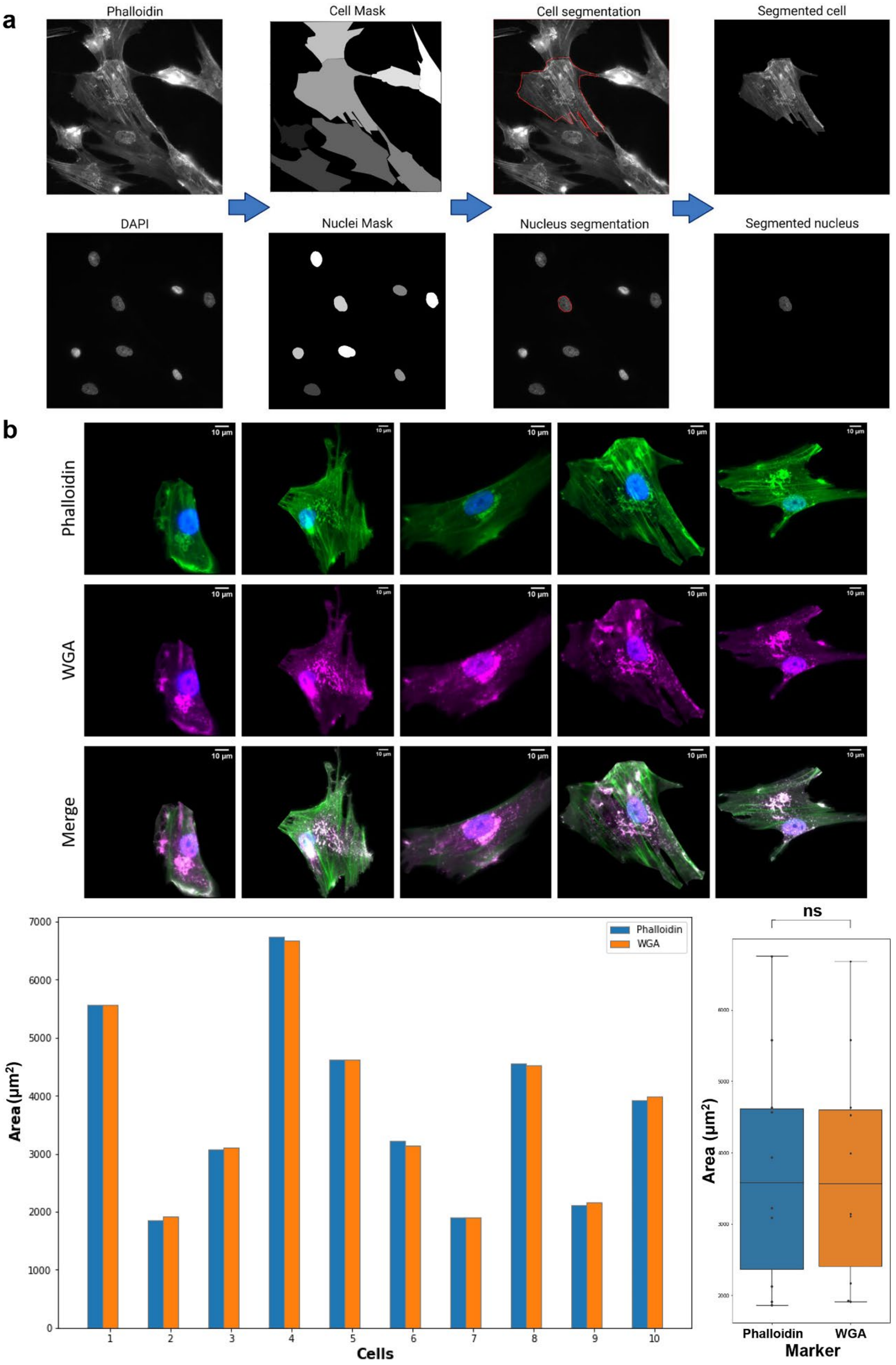

a
Phalloidin
Cell Mask
Cell segmentation
Segmented cell
DAPI
Nuclei Mask
Nucleus segmentation
Segmented nucleus
b
Phalloidin
WGA
Merge
10 µm
Phalloidin
WGA
Area (µm²)
Cells
ns
Phalloidin
WGA
Marker
Area (µm²)

Supplementary Figure 10. Segmentation of single cells.

(a) Segmentation of single cells using Phalloidin as the cell mask (top row) and DAPI as the nuclear mask (bottom row). Column 1: raw signal from Phalloidin or DAPI, Column 2: cell or nuclear mask label image from the raw signal, Column 3: cell or nuclear segmentation boundary example from the masks, Column 4: resulting segmented cell and its nucleus.

(b) Comparing the area obtained by segmenting single cells (n=10) using two markers - Phalloidin (green, row 1) and WGA (magenta, row 2). The bottom bar graph shows the sum intensities of 10 image regions. The Mann-Whitney test shows no statistical significance between the areas of the two markers (p=0.85), indicating that the area distributions are the same for the two markers and either of them can be used for cell segmentation. All scale bars 10 µm.

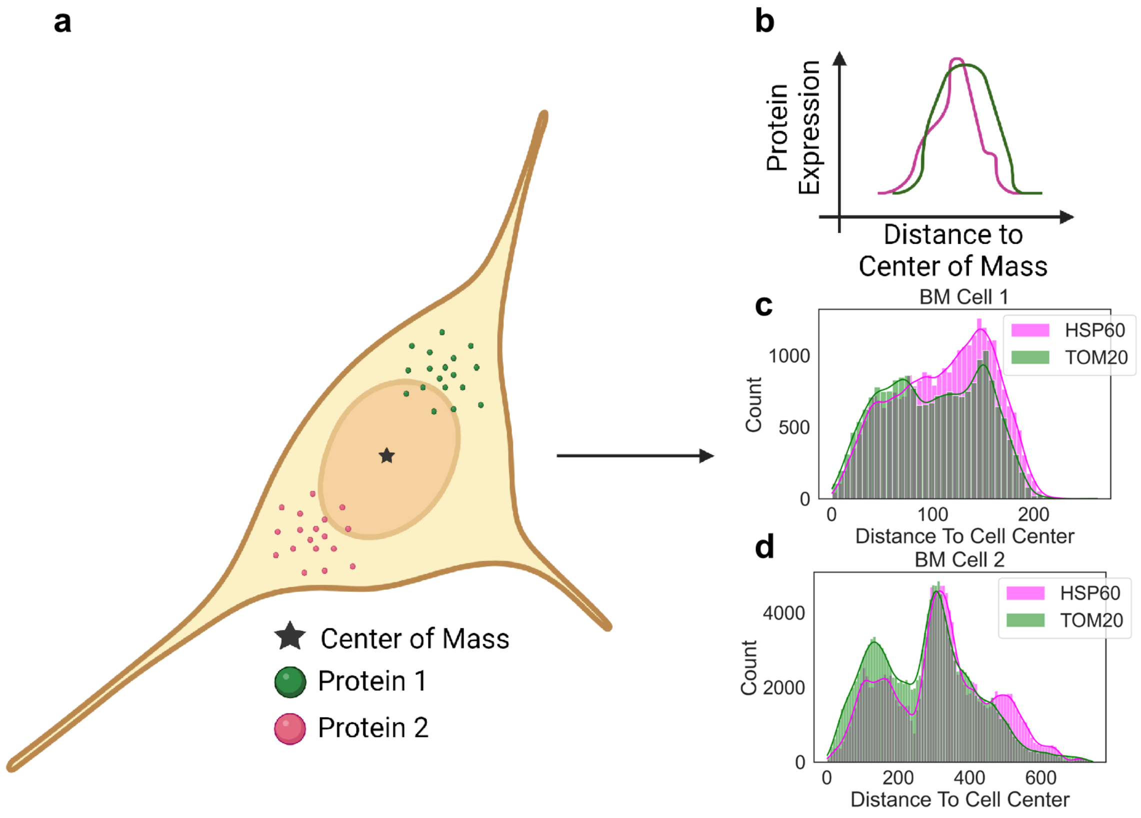

Supplementary Figure 11. Conversion of 2D spatial coordinates to 1D spatial distributions for KS Hypothesis Testing.

(a) Example illustration of 2D cell focusing on two arbitrary protein markers: green and magenta. The black star denotes the cell's center of mass computed from the cell segmentation mask. Created with BioRender.com.

(b) Calculated distances from each protein (green, magenta) pixel to the center of mass resulting in 1D histograms of spatial distances. KS Hypothesis Testing determines if the two signals (green and magenta) come from significantly different underlying distributions. Created with BioRender.com.

(c) Example BM cell 1 with significantly (p-value ≤ 0.0001) different underlying distributions for the target mitochondria organelle labeled by HSP60 (magenta) and TOM20 (green) protein markers. See **Fig. 4d** for complete cells.

(d) Example BM cell 2 with no significant statistical difference in mitochondria spatial expression between HSP60 (magenta) and TOM20 (green) protein markers. See **Fig. 4d** for complete cells.

Marker	Target	Function
HSP60	Mitochondria	Heat Shock Protein - molecular chaperone; aids protein folding; essential for the protein import into the mitochondrial matrix [1–3]
TOM20	Mitochondria	Translocase of the outer membrane of mitochondria; assists the movement of nucleus-encoded precursor proteins [4,5]
ATF6	Endoplasmic Reticulum	Activating transcription factor 6 - Acts during ER stress by activating unfolding protein response (UPR) target genes [6,7]
GOLPH4/ GPP130	Golgi apparatus	Golgi integral membrane protein. Plays a role in endosome to Golgi protein trafficking [8,9]
Sortilin	Localized to membranes of the Golgi, ER, nucleus, endosomes, and lysosomes.	Sorting receptor in the Golgi compartment. Clearance receptor on the cell surface. Regulates intracellular protein transport through secretory or endocytic pathways [10,11]
Concanavalin A	Endoplasmic reticulum	Selectively binds to a-mannopyranosyl and a-glucopyranosyl residues found in the mammalian cell membrane [12,13]
Phalloidin	F-Actin	Binds to filamentous actin (F-actin) which takes part in various cellular functions (cell motility, maintenance of cell shape, and regulation of transcription) [13,14]
WGA	Golgi apparatus and plasma membrane	Carbohydrate-binding lectin binding to sialic acid and N-acetylglucosamine residues of glycoproteins. Labels the cell membrane of mammalian cells and tissues. Also stains multiple proteins and lipids in the Golgi that are glycosylated [13,15]
Nucleolin	Nucleolus	Plays a role in ribosome biogenesis and rRNA synthesis [16]
β-Tubulin	Cytosol	One of the forms of Tubulin is a cytoskeleton component. Multiple β-Tubulin isoforms are expressed in different regions of the cell [17]

Supplementary Table 1. Description of antibodies used in 10-plex organelle mapping in MSCs.

Cycle	Channel	Antibody	Dilution
1	 488<br>647	DAPI<br>Sortilin<br>β-Tubulin	1:1000<br>1:100<br>1:200
2	 555<br>647	DAPI<br>Phalloidin<br>WGA	1:1000<br>1:40<br>1:400
3	 647	DAPI<br>ATF6	1:1000<br>1:200
4	 647	DAPI<br>TOM20	1:1000<br>1:200
5	 647	DAPI<br>GOLPH4	1:1000<br>1:500
6	 647	DAPI<br>HSP60	1:1000<br>1:200
7	 647	DAPI<br>Nucleolin	1:1000<br>1:250
8	 488	DAPI<br>Concanavalin A	1:1000<br>1:20

Supplementary Table 2. Order of antibodies used for rapid multiplexed immunofluorescence of organelle markers in MSCs.

Markers	BM MSC				UC MSC				BM vs UC	
	Min	Mean	Median	Max	Min	Mean	Median	Max	P_val	U_stat
**DAPI_GOLPH4**	-0.39019	0.062123	0.074778	0.317464	-0.09886	0.105488	0.0842	0.386037	0.576613	120
**DAPI_HSP60**	-0.42051	0.002756	-0.01028	0.376206	-0.23394	0.022768	0.008788	0.280216	0.806636	73
**DAPI_Nucleolin**	0.543317	0.875238	0.902931	0.975774	0.367504	0.673049	0.699571	0.883398	3.85E-05	322
**DAPI_Phalloidin**	0.216938	0.382776	0.348025	0.619993	0.488149	0.612673	0.625872	0.674625	0.000594	6
**DAPI_Sortilin**	0.325445	0.621453	0.654387	0.880571	0.17685	0.426505	0.394083	0.642554	0.003707	161
**DAPI_TOM20**	-0.35485	0.11469	0.074217	0.697917	-0.16003	0.098451	0.056604	0.390662	0.797229	127
**DAPI_WGA**	0.347376	0.516248	0.484481	0.720834	0.529252	0.661049	0.687087	0.740772	0.029309	9
**Nucleolin_Phalloidin**	0.194735	0.314293	0.29751	0.590012	0.343967	0.466714	0.449239	0.686738	0.006085	12
**Nucleolin_Sortilin**	0.267203	0.629767	0.667747	0.890442	0.00239	0.312953	0.299362	0.608249	0.000317	188
**Nucleolin_TOM20**	-0.34576	0.088968	0.038012	0.680387	-0.1461	0.121276	0.096396	0.448528	0.755208	104
**Nucleolin_WGA**	0.218807	0.436256	0.407739	0.633647	0.423519	0.497395	0.481296	0.603469	0.316792	22
**BetaTubulin_ConcanavalinA**	0.496492	0.691746	0.7405	0.789489	0.413759	0.573477	0.498921	0.791367	0.412698	14
**BetaTubulin_DAPI**	-0.31142	0.139395	0.108773	0.625378	-0.06784	0.228074	0.261448	0.473662	0.203879	63
**BetaTubulin_GOLPH4**	0.283838	0.574685	0.568009	0.806661	0.137294	0.393023	0.328376	0.714282	0.021868	124
**BetaTubulin_HSP60**	0.327023	0.67031	0.717532	0.865904	0.16363	0.409397	0.410274	0.60755	0.007184	73
**BetaTubulin_Nucleolin**	-0.3023	0.112284	0.097697	0.632351	-0.0552	0.147151	0.156322	0.37754	0.633368	75
**BetaTubulin_Phalloidin**	0.244745	0.626865	0.672226	0.867982	0.494649	0.582824	0.528044	0.780561	0.720915	32
**BetaTubulin_Sortilin**	0.307572	0.597394	0.597909	0.907544	0.124691	0.468492	0.458365	0.75202	0.069825	83

**BetaTubulin_TOM20**	0.576419	0.772853	0.802629	0.92124	0.113598	0.409049	0.38787	0.678083	0.000138	124
**BetaTubulin_WGA**	0.428599	0.648152	0.696771	0.853219	0.365344	0.509655	0.521973	0.641648	0.197059	32
**Phalloidin_Sortilin**	0.338837	0.538326	0.546553	0.740659	0.547864	0.712553	0.722694	0.860582	0.031219	10
**Phalloidin_TOM20**	0.100105	0.536913	0.592766	0.793341	0.173488	0.528836	0.564509	0.763669	0.963585	35
**Phalloidin_WGA**	0.511922	0.820922	0.855109	0.963523	0.812238	0.88441	0.919115	0.921877	0.357895	16
**ATF6_BetaTubulin**	0.230635	0.638931	0.651492	0.831835	0.111629	0.527598	0.532653	0.833342	0.074116	101
**ATF6_ConcanavalinA**	0.537429	0.60885	0.606558	0.684853	0.400188	0.691459	0.721718	0.858331	0.460606	11
**ATF6_DAPI**	-0.37689	0.07511	0.079387	0.479719	0.23692	0.464934	0.469409	0.695617	9.47E-06	12
**ATF6_GOLPH4**	0.438384	0.654637	0.655356	0.871873	0.207702	0.448072	0.41293	0.74775	0.000512	220
**ATF6_HSP60**	0.288369	0.595477	0.621389	0.770715	0.343904	0.549817	0.575758	0.741718	0.402504	87
**ATF6_Nucleolin**	-0.37893	0.053693	0.004319	0.46994	0.078385	0.35306	0.327777	0.641588	0.000504	37
**ATF6_Phalloidin**	0.125327	0.481911	0.549689	0.712088	0.684505	0.792287	0.79503	0.891105	0.000646	2
**ATF6_Sortilin**	0.11899	0.421913	0.408688	0.654826	0.633488	0.766087	0.757086	0.878789	2.62E-05	1
**ATF6_TOM20**	0.559568	0.652566	0.634778	0.822792	0.311763	0.584473	0.565917	0.810053	0.117297	132
**ATF6_WGA**	0.252876	0.473902	0.383127	0.730323	0.699616	0.753623	0.725782	0.863312	0.010256	3
**ConcanavalinA_DAPI**	-0.05448	0.303461	0.362518	0.543288	0.000862	0.222493	0.181937	0.550333	0.604196	22
**ConcanavalinA_GOLPH4**	0.464922	0.57005	0.532831	0.749616	0.544043	0.689883	0.696733	0.816394	0.198601	9
**ConcanavalinA_HSP60**	0.485728	0.610834	0.568922	0.819765	0.588811	0.688966	0.684291	0.785184	0.367677	10
**ConcanavalinA_Nucleolin**	-0.03387	0.299571	0.335504	0.561149	0.011404	0.216478	0.205355	0.577092	0.682828	19
**ConcanavalinA_Phalloidin**	0.309975	0.533999	0.533309	0.759403	0.47781	0.732062	0.744397	0.911059	0.171429	5
**ConcanavalinA_Sortilin**	0.610423	0.641146	0.643976	0.666209	0.356439	0.604905	0.62096	0.864761	1	16

**ConcanavalinA_TOM20**	0.569595	0.64355	0.611876	0.780851	0.560793	0.719975	0.720785	0.823378	0.282828	9
**ConcanavalinA_WGA**	0.433955	0.621926	0.607835	0.83808	0.546859	0.660384	0.609458	0.824837	1	6
**GOLPH4_HSP60**	0.385765	0.601535	0.651802	0.790159	0.341387	0.558922	0.574245	0.73119	0.370844	88
**GOLPH4_Nucleolin**	-0.3879	0.044443	0.036952	0.267336	-0.10264	0.097832	0.054791	0.413396	0.55227	119
**GOLPH4_Phalloidin**	0.08783	0.479072	0.525426	0.731147	0.224822	0.513823	0.533916	0.759271	0.820082	33
**GOLPH4_Sortilin**	0.043945	0.366134	0.370333	0.68059	0.199859	0.388441	0.352215	0.682184	0.979483	83
**GOLPH4_TOM20**	0.306794	0.599249	0.593468	0.803896	0.390917	0.570744	0.586689	0.722106	0.555735	119
**GOLPH4_WGA**	0.152016	0.4553	0.49672	0.727791	0.330933	0.434106	0.403628	0.598236	0.870588	28
**HSP60_Nucleolin**	-0.41367	-0.02945	-0.07352	0.334086	-0.18366	0.048875	-0.00455	0.332282	0.417302	57
**HSP60_Phalloidin**	0.265471	0.523268	0.450504	0.773128	0.286359	0.500554	0.541214	0.683799	0.949717	25
**HSP60_Sortilin**	0.152477	0.52596	0.540497	0.753786	0.237453	0.494478	0.487629	0.694411	0.599361	69
**HSP60_TOM20**	0.575209	0.705255	0.718745	0.827389	0.553234	0.681668	0.680737	0.797218	0.602068	81
**HSP60_WGA**	0.313627	0.527848	0.486146	0.795322	0.262485	0.404339	0.323262	0.627269	0.278788	18
**Sortilin_TOM20**	0.223946	0.542002	0.525151	0.889835	0.223506	0.492665	0.467866	0.728453	0.55876	76
**Sortilin_WGA**	0.514945	0.656143	0.663811	0.80966	0.571951	0.666756	0.636943	0.791373	1	16
**TOM20_WGA**	0.30527	0.557022	0.545052	0.809731	0.298807	0.458673	0.405488	0.724908	0.379121	32

Supplementary Table 3. Minimum, mean, median, and maximum of Pearson's correlation for all marker pairs for BM and UC MSCs, with the p-value obtained from the Mann-Whitney test (corresponding to Figure 3A).

Markers	BM MSC				UC MSC				BM vs UC	
	Min	Mean	Median	Max	Min	Mean	Median	Max	P_val	U_stat
**DAPI_GOLPH4**	0.029933	0.140341	0.110874	0.612356	0.038534	0.205634	0.129603	0.546821	0.33979	109
**DAPI_HSP60**	0.032031	0.107235	0.104096	0.206923	0.025207	0.202978	0.128565	0.508919	0.567915	67
**DAPI_Nucleolin**	0.095349	0.217035	0.172187	0.701208	0.089205	0.238682	0.197883	0.53368	0.501915	157
**DAPI_Phalloidin**	0.068819	0.205241	0.150994	0.686085	0.195755	0.349284	0.328462	0.522633	0.012561	16
**DAPI_Sortilin**	0.080312	0.189254	0.15417	0.688467	0.07763	0.228077	0.179385	0.494266	0.461095	81
**DAPI_TOM20**	0.031419	0.161131	0.12788	0.680571	0.047601	0.218104	0.148579	0.544187	0.593595	106
**DAPI_WGA**	0.082669	0.221316	0.170709	0.686617	0.201016	0.301321	0.277963	0.448344	0.049948	11
**Nucleolin_Phalloidin**	0.08118	0.237942	0.184388	0.721285	0.183496	0.339912	0.324401	0.51136	0.177503	29
**Nucleolin_Sortilin**	0.100414	0.22385	0.190346	0.720713	0.074958	0.224461	0.17072	0.480421	0.526843	120
**Nucleolin_TOM20**	0.050427	0.186549	0.159006	0.701684	0.067648	0.220288	0.143254	0.538955	0.983417	111
**Nucleolin_WGA**	0.102003	0.255952	0.19887	0.721176	0.186831	0.291371	0.257728	0.463197	0.361571	23
**BetaTubulin_ConcanavalinA**	0.16241	0.18445	0.171137	0.233117	0.095782	0.189548	0.108706	0.522169	0.190476	16
**BetaTubulin_DAPI**	0.038165	0.180007	0.122648	0.68698	0.041233	0.153385	0.108799	0.49191	0.548953	103
**BetaTubulin_GOLPH4**	0.085206	0.198218	0.176466	0.6367	0.053937	0.149621	0.09658	0.509404	0.042445	119
**BetaTubulin_HSP60**	0.120676	0.181837	0.167848	0.294287	0.05642	0.148507	0.10222	0.492257	0.012979	71
**BetaTubulin_Nucleolin**	0.063182	0.223111	0.169347	0.72395	0.038043	0.149407	0.103773	0.487062	0.125672	116
**BetaTubulin_Phalloidin**	0.052337	0.236681	0.156967	0.728057	0.107428	0.215013	0.123001	0.506623	0.277124	39

**BetaTubulin_Sortilin**	0.118466	0.241298	0.204575	0.732019	0.050634	0.152346	0.114866	0.527398	0.003803	97
**BetaTubulin_TOM20**	0.136339	0.232811	0.193219	0.695106	0.054592	0.154842	0.09975	0.473625	0.021489	100
**BetaTubulin_WGA**	0.094124	0.265419	0.191987	0.728109	0.101372	0.180874	0.119545	0.321705	0.432353	28
**Phalloidin_Sortilin**	0.077295	0.224957	0.171793	0.731656	0.173095	0.33182	0.325545	0.496152	0.093407	14
**Phalloidin_TOM20**	0.059375	0.200287	0.155203	0.69615	0.101767	0.316583	0.325452	0.520102	0.493644	28
**Phalloidin_WGA**	0.127441	0.294336	0.231962	0.782358	0.231311	0.328896	0.235917	0.519462	0.545614	19
**ATF6_BetaTubulin**	0.07713	0.206393	0.177691	0.663276	0.049964	0.17242	0.117646	0.535966	0.095159	99
**ATF6_ConcanavalinA**	0.140418	0.16471	0.15982	0.198783	0.136196	0.299043	0.213225	0.551848	0.460606	11
**ATF6_DAPI**	0.039889	0.145055	0.111989	0.616466	0.078378	0.243452	0.195032	0.524616	0.008761	62
**ATF6_GOLPH4**	0.108655	0.196016	0.170058	0.637351	0.072018	0.213853	0.135709	0.529135	0.406098	150
**ATF6_HSP60**	0.104016	0.164404	0.157385	0.29108	0.06696	0.235376	0.151047	0.510431	0.885234	69
**ATF6_Nucleolin**	0.046195	0.170277	0.146255	0.637667	0.08512	0.235627	0.192502	0.515698	0.077286	84
**ATF6_Phalloidin**	0.050772	0.186974	0.142172	0.648206	0.191566	0.349565	0.341216	0.535523	0.027311	11
**ATF6_Sortilin**	0.084176	0.161247	0.151105	0.290217	0.103548	0.257465	0.202064	0.510202	0.069294	47
**ATF6_TOM20**	0.110376	0.173772	0.169589	0.293165	0.091216	0.250017	0.181488	0.514897	0.890085	94
**ATF6_WGA**	0.084178	0.208003	0.157136	0.64311	0.197514	0.312	0.272604	0.50528	0.077656	8
**ConcanavalinA_DAPI**	0.068039	0.134797	0.14854	0.174069	0.010144	0.250507	0.15323	0.52669	0.71049	15
**ConcanavalinA_GOLPH4**	0.116307	0.158268	0.166661	0.183443	0.098642	0.28318	0.190439	0.537021	0.26014	10
**ConcanavalinA_HSP60**	0.128374	0.179046	0.182393	0.223023	0.112997	0.285997	0.195008	0.540085	0.682828	13

**ConcanavalinA_Nucleolin**	0.086394	0.151197	0.166215	0.185966	0.126744	0.286909	0.195202	0.523105	0.460606	11
**ConcanavalinA_Phalloidin**	0.056748	0.138347	0.155869	0.184902	0.150698	0.347229	0.33882	0.55311	0.171429	5
**ConcanavalinA_Sortilin**	0.138492	0.195578	0.199164	0.245491	0.130307	0.277163	0.178028	0.496645	1	16
**ConcanavalinA_TOM20**	0.137467	0.175938	0.182917	0.200449	0.123607	0.300654	0.205623	0.533969	0.569697	12
**ConcanavalinA_WGA**	0.092071	0.174946	0.20021	0.207293	0.145763	0.286049	0.198647	0.513738	1	6
**GOLPH4_HSP60**	0.099651	0.159818	0.163529	0.23327	0.068394	0.241964	0.129313	0.522892	0.750832	78
**GOLPH4_Nucleolin**	0.038254	0.166481	0.140757	0.627265	0.057044	0.207773	0.137589	0.536126	0.842951	130
**GOLPH4_Phalloidin**	0.049873	0.183734	0.145713	0.638622	0.111599	0.31445	0.307422	0.520862	0.437083	27
**GOLPH4_Sortilin**	0.073334	0.153295	0.142385	0.265553	0.070501	0.208047	0.11778	0.496356	0.625106	94
**GOLPH4_TOM20**	0.099973	0.165855	0.170396	0.23814	0.073102	0.24121	0.171869	0.555621	0.878593	101
**GOLPH4_WGA**	0.068093	0.204101	0.163804	0.634983	0.107153	0.268693	0.245805	0.476008	0.548739	20
**HSP60_Nucleolin**	0.045298	0.130364	0.128896	0.273187	0.049015	0.198623	0.106824	0.506065	0.862015	75
**HSP60_Phalloidin**	0.061138	0.152704	0.15024	0.259035	0.102167	0.3105	0.298625	0.524778	0.344988	16
**HSP60_Sortilin**	0.104884	0.179364	0.159302	0.304518	0.063344	0.234236	0.140627	0.505232	0.511406	71
**HSP60_TOM20**	0.116491	0.179458	0.174055	0.292918	0.081971	0.242916	0.165204	0.517226	0.862015	75
**HSP60_WGA**	0.083358	0.169871	0.165588	0.276312	0.098521	0.240743	0.170423	0.453287	0.921212	11
**Sortilin_TOM20**	0.104367	0.224192	0.186117	0.694328	0.085092	0.235964	0.152351	0.491404	0.600878	75
**Sortilin_WGA**	0.116207	0.255394	0.212912	0.734139	0.175377	0.278567	0.188996	0.471329	0.884615	18
**TOM20_WGA**	0.093864	0.220697	0.176838	0.696823	0.09784	0.287557	0.27248	0.507427	0.446154	17

Supplementary Table 4. Minimum, mean, median, and maximum colocalization for all marker pairs for BM and UC MSCs, with the p-value obtained from the Mann-Whitney test (corresponding to Figure 3B).

Marker	BM MSC				UC MSC				BM vs UC	
	Min	Mean	Median	Max	Min	Mean	Median	Max	P_val	U_stat
**ATF6**	95613	251152.3	182058	540950	74862	354998.1	297711	919540	0.071296	90
**BetaTubulin**	95613	246384.8	187415	540950	192623	318666.6	272743	603029	0.08532	57
**ConcanavalinA**	125253	283039.8	258858.5	489189	62483	252557.1	248294	470513	0.825175	20
**DAPI**	95613	272233.7	248057	540950	62483	360352	297891	919540	0.114574	149
**GOLPH4**	95613	243617.6	182058	540950	62483	321317.4	272743	919540	0.170779	111
**HSP60**	95613	249348.2	184736.5	540950	62483	297553.1	293154.5	603029	0.425315	68
**Nucleolin**	95613	267434.1	236405	540950	74862	360970.9	297891	919540	0.096196	138
**Phalloidin**	95613	278803.2	274030	489189	117910	294907.7	293154.5	470513	0.922539	52
**Sortilin**	95613	258700.6	215057.5	540950	117910	362750.6	297711	919540	0.101707	67
**TOM20**	95613	235855.4	187415	489189	62483	309172.9	288598	603029	0.174001	91
**WGA**	95613	268758.1	248110	489189	192623	319911.5	308255	470513	0.484484	27

Supplementary Table 5. Minimum, mean, median, and maximum area for all markers for BM and UC MSCs, with the p-value obtained from the Mann-Whitney test (corresponding to Figure 3C).

Marker	BM MSC				UC MSC				BM vs UC	
	Min	Mean	Median	Max	Min	Mean	Median	Max	P_val	U_stat
**ATF6**	33.17142	98.03184	100.1086	145.7242	40.8177	91.76635	81.2978	185.1369	0.071296	90
**BetaTubulin**	31.58946	91.9572	94.03891	145.7242	40.8177	104.4021	93.14664	185.1369	0.08532	57
**ConcanavalinA**	86.07068	98.85573	95.53423	118.2838	40.8177	78.65693	81.2978	127.5041	0.825175	20
**DAPI**	31.58946	92.09201	94.03891	145.7242	40.8177	94.06361	84.7404	185.1369	0.114574	149
**GOLPH4**	33.17142	95.59522	94.61209	145.7242	40.8177	92.95852	84.7404	185.1369	0.170779	111
**HSP60**	71.59402	103.3321	103.6725	145.7242	40.8177	97.97317	103.5249	177.7374	0.425315	68
**Nucleolin**	31.58946	89.83544	90.75636	145.7242	40.8177	93.85647	84.7404	185.1369	0.096196	138
**Phalloidin**	31.58946	82.7879	88.31179	118.2838	40.8177	65.02848	60.97885	98.11028	0.922539	52

**Sortilin**	31.58946	94.8242	95.05421	145.7242	40.8177	94.89408	88.183	185.1369	0.101707	67
**TOM20**	31.58946	96.15309	94.61209	145.7242	40.8177	95.46001	88.183	185.1369	0.174001	91
**WGA**	31.58946	81.12397	85.34437	118.2838	42.43858	69.56205	68.84967	98.11028	0.484484	27

Supplementary Table 6. Minimum, mean, median, and maximum intensity for all markers for BM and UC MSCs, with the p-value obtained from the Mann-Whitney test (corresponding to Figure 3D).

Marker Pair	BM1	BM2	BM3	BM4	BM5	BM6	BM7	BM Average	BM StdDev
ATF6_DAPI	0.196423	0.044327	0.092174	0.101055	0.290203	0.084216	0.036656	0.120722	0.091189
ATF6_GOLPH4	0.886905	0.743921	0.736341	0.746596	0.684851	0.625186	0.474311	0.69973	0.127224
ATF6_Nucleolin	0.16351	0.003114	0.063636	0.091991	0.249984	0.071487	0.035529	0.097036	0.083875
ATF6_TOM20	0.830419	0.638131	0.704807	0.716485	0.709677	0.640397	0.625479	0.695056	0.070899
DAPI_GOLPH4	0.258731	-0.01459	0.032707	0.102173	0.326128	0.030444	0.002435	0.105432	0.134254
DAPI_Nucleolin	0.900525	0.552446	0.927836	0.949862	0.90688	0.888909	0.956599	0.869008	0.141837
DAPI_TOM20	0.165633	0.047519	0.170235	0.126379	0.25699	0.070878	0.083174	0.131544	0.07246
GOLPH4_Nucleolin	0.218046	-0.01295	0.012693	0.084748	0.244019	0.0105	-0.00011	0.079564	0.108318
GOLPH4_TOM20	0.785866	0.52624	0.64621	0.776565	0.675298	0.616704	0.358623	0.626501	0.148656
Nucleolin_TOM20	0.138058	0.008803	0.1439	0.108819	0.191075	0.062622	0.084286	0.105366	0.059871
Marker Pair	UC1	UC2	UC3	UC4	UC5	UC6	UC7	UC Average	UC StdDev
ATF6_DAPI	0.407033	0.335367	0.578863	0.517145	0.41034	0.624892	0.398562	0.467457	0.107062
ATF6_GOLPH4	0.572879	0.297727	0.651611	0.430775	0.27216	0.494814	0.344256	0.437746	0.143218
ATF6_Nucleolin	0.36727	0.349333	0.583028	0.382364	0.110109	0.374694	0.123962	0.327251	0.16395
ATF6_TOM20	0.808696	0.688347	0.801106	0.482706	0.392987	0.756106	0.621493	0.650206	0.161039
DAPI_GOLPH4	0.114759	0.056249	0.205999	0.058438	-0.00208	0.362382	0.058118	0.121981	0.124319
DAPI_Nucleolin	0.875816	0.884637	0.837625	0.726613	0.375265	0.575322	0.503603	0.682697	0.200883
DAPI_TOM20	0.217138	0.026181	0.28817	0.067537	0.011137	0.446522	0.10885	0.166505	0.159544
GOLPH4_Nucleolin	0.109928	0.066897	0.29133	0.030311	-0.008	0.210649	-0.00062	0.100072	0.113055
GOLPH4_TOM20	0.575517	0.299882	0.659152	0.678319	0.480347	0.511235	0.439772	0.520603	0.131685
Nucleolin_TOM20	0.207655	0.077855	0.352141	0.004792	-0.00877	0.258247	0.002558	0.127783	0.144732

Supplementary Table 7. Pearson's correlation coefficient values for selected marker pairs for 7 BM and UC MSCs (corresponding to Figure 4A).

Marker Pair	BM1	BM2	BM3	BM4	BM5	BM6	BM7	BM Average	BM StdDev
DAPI_ATF6	0.198657	0.039487	0.086898	0.093114	0.171108	0.067664	0.067568	0.103499	0.058711
DAPI_GOLPH4	0.213441	0.018068	0.049052	0.09539	0.189666	0.035477	0.0349	0.090856	0.07964
DAPI_Nucleolin	0.781629	0.432631	0.922132	0.944076	0.818159	0.871556	0.963145	0.819047	0.182802
GOLPH4_ATF6	0.765817	0.618961	0.505556	0.597201	0.525747	0.468361	0.391373	0.553288	0.121021
GOLPH4_DAPI	0.213441	0.018068	0.049052	0.09539	0.189666	0.035477	0.0349	0.090856	0.07964
GOLPH4_Nucleolin	0.203745	0.008078	0.039074	0.091449	0.153867	0.027703	0.033591	0.079644	0.073752
Nucleolin_ATF6	0.185392	0.013872	0.074979	0.090165	0.141186	0.05908	0.066248	0.090132	0.056658
Nucleolin_DAPI	0.781629	0.432631	0.922132	0.944076	0.818159	0.871556	0.963145	0.819047	0.182802
Nucleolin_GOLPH4	0.203745	0.008078	0.039074	0.091449	0.153867	0.027703	0.033591	0.079644	0.073752
TOM20_ATF6	0.713756	0.522471	0.444103	0.569148	0.48106	0.460904	0.446174	0.519659	0.096766
TOM20_DAPI	0.175406	0.038659	0.092029	0.109527	0.217628	0.059561	0.079293	0.1103	0.06424
TOM20_GOLPH4	0.636476	0.491008	0.423903	0.651613	0.48217	0.437755	0.273494	0.485203	0.130031
TOM20_Nucleolin	0.165328	0.016058	0.08344	0.105156	0.172778	0.050871	0.078637	0.096039	0.057259
Marker Pair	UC1	UC2	UC3	UC4	UC5	UC6	UC7	UC Average	UC StdDev
DAPI_ATF6	0.196808	0.092944	0.285006	0.271964	0.152621	0.290183	0.171751	0.208754	0.075846
DAPI_GOLPH4	0.07593	0.029461	0.183259	0.086865	0.014359	0.231524	0.044002	0.095057	0.081912
DAPI_Nucleolin	0.741429	0.761125	0.708505	0.665065	0.164178	0.370388	0.279771	0.527209	0.248358
GOLPH4_ATF6	0.480053	0.303715	0.619116	0.419372	0.262292	0.418901	0.289242	0.398956	0.126258
GOLPH4_DAPI	0.07593	0.029461	0.183259	0.086865	0.014359	0.231524	0.044002	0.095057	0.081912
GOLPH4_Nucleolin	0.071209	0.040352	0.236832	0.066246	0.000957	0.093926	0.006353	0.073696	0.079605
Nucleolin_ATF6	0.173156	0.110506	0.324521	0.210464	0.024332	0.109092	0.041439	0.14193	0.104087
Nucleolin_DAPI	0.741429	0.761125	0.708505	0.665065	0.164178	0.370388	0.279771	0.527209	0.248358

**Nucleolin_GOLPH4**	0.071209	0.040352	0.236832	0.066246	0.000957	0.093926	0.006353	0.073696	0.079605
**TOM20_ATF6**	0.670208	0.56118	0.709138	0.455341	0.35816	0.642097	0.492202	0.555475	0.127329
**TOM20_DAPI**	0.13406	0.025831	0.246479	0.077721	0.023136	0.27258	0.077346	0.12245	0.10107
**TOM20_GOLPH4**	0.477261	0.30591	0.59053	0.585311	0.353276	0.419717	0.321136	0.436163	0.119095
**TOM20_Nucleolin**	0.127179	0.0478	0.291461	0.051048	0.001147	0.111043	0.007882	0.09108	0.100283

Supplementary Table 8. Colocalization values for selected marker pairs for 7 BM and UC MSCs (corresponding to Figure 4B).

Marker Pair	BM1	BM2	BM3	BM4	BM5	BM6	BM7
**ATF6**	72077	53496	26147	92562	91513	137280	79126
**BetaTubulin**	110718	43207	29110	137453	118420	162026	177176
**ConcanavalinA**	74479	58762	30292	147074	103561	167414	118167
**DAPI**	18000	16198	10203	21401	26186	21884	24240
**GOLPH4**	73537	44528	29841	86267	50304	127784	89119
**HSP60**	106731	47291	43028	133622	92454	231791	156379
**Nucleolin**	2158	1765	2572	1027	2498	887	707
**Phalloidin**	116287	55108	36672	266663	174239	229626	289824
**Sortilin**	61584	35704	31165	88538	76510	180883	52895
**TOM20**	88060	49251	28398	160884	173177	185333	137833
**WGA**	90379	50818	34451	138099	96925	231986	134474
**Marker Pair**	**UC1**	**UC2**	**UC3**	**UC4**	**UC5**	**UC6**	**UC7**
**ATF6**	95622	64911	64766	95730	40275	85629	254557
**BetaTubulin**	198615	148244	154339	327964	208441	390137	664577
**ConcanavalinA**	119233	88994	105803	191096	78745	140352	295241
**DAPI**	25724	23470	24846	23510	19394	26163	35329
**GOLPH4**	105132	75821	69107	24267	31549	76274	77424
**HSP60**	247370	125875	152705	348499	114527	298772	488934
**Nucleolin**	10989	9814	3180	3860	2257	338	13433
**Phalloidin**	145333	71630	76489	159617	53867	238990	301581
**Sortilin**	178156	89586	70557	231671	88344	112341	413930
**TOM20**	122737	75906	101154	116887	65806	124685	311985
**WGA**	130533	83281	83649	110234	60433	110821	245917

Supplementary Table 9. Area of selected markers (in square pixels) for 7 BM and UC MSCs obtained from CellProfiler (corresponding to Figure 4C).

Markers	BM1	BM2	BM3	BM4	BM5	BM6	BM7
**ATF6**	176.3	174.7	170.49	346.84	365.07	364.79	204.44
**BetaTubulin**	242.35	152.8	182.2	408.64	429.67	383.02	278.02
**ConcanavalinA**	170.62	176.34	180.06	408.64	364.21	376.87	247.69
**DAPI**	112.56	106.95	108.17	136.3	162.94	141.53	134.99
**GOLPH4**	176.07	165.82	177.4	350.53	206.8	368.23	213.95
**HSP60**	262.32	164.47	231.04	358.46	413.57	415.93	267.94
**Nucleolin**	49.68	43.63	54.67	31.32	49.68	31.36	22.39
**Phalloidin**	294.52	189.62	205.06	483.99	529.59	450.02	392.95
**Sortilin**	168.3	139.98	194.53	253.56	286.93	416.2	183.64
**TOM20**	194.56	167.36	187.51	449.22	571.29	418.18	249.73
**WGA**	205.62	190.17	203.32	477.82	343.91	427.62	338.99
**Marker Pair**	**UC1**	**UC2**	**UC3**	**UC4**	**UC5**	**UC6**	**UC7**
**ATF6**	185.82	212.52	214.96	245.46	200	269.98	408.79
**BetaTubulin**	265.05	266.07	290.35	459.55	407.43	725.51	784.57
**ConcanavalinA**	213.64	230.11	239.23	294.85	292.19	337.3	591.77
**DAPI**	137.03	137.16	141.49	138.21	122.38	141.31	162.41
**GOLPH4**	232.78	221.8	172.72	141.47	159.68	281.32	344.52
**HSP60**	276.84	254.91	293.33	544.46	315.08	687.99	668.44
**Nucleolin**	88.48	101.56	76.11	59.14	49.53	17.05	134.58
**Phalloidin**	222.84	215.17	211.21	284.31	235.53	660.83	651.93
**Sortilin**	262.86	245.3	250.9	428.94	288.3	325.11	596.86
**TOM20**	196.72	226.27	224.92	296.1	248.79	319.57	520.81
**WGA**	212.29	232.73	240.43	249.75	247.39	289.91	473.24

Supplementary Table 10. Minor axis length (in pixels) of selected markers for 7 BM and UC MSCs obtained from CellProfiler (corresponding to Figure 4C).

Markers	BM1	BM2	BM3	BM4	BM5	BM6	BM7
**ATF6**	636.77	468.73	205	561.16	452.47	615.57	541.14
**BetaTubulin**	786.37	439.98	211.33	730.36	584.37	814.98	978.95
**ConcanavalinA**	640.94	506.47	223.28	761.49	528.2	820.95	720.3
**DAPI**	208.99	195.66	121.24	200.28	205	197.94	229.06
**GOLPH4**	622.37	419.11	223.93	517.1	396.7	657.58	597.29
**HSP60**	770.53	413.77	268.36	595.57	522.41	1021.36	905.55
**Nucleolin**	62.28	54.54	82.92	45.92	84.07	38.14	74.5
**Phalloidin**	778.68	466.74	246.71	930.7	648.23	1033.74	1100.64
**Sortilin**	597.71	365.05	210.77	557.17	423.54	888.56	445.49
**TOM20**	684.36	426.75	201.95	699.62	720.06	777.22	882.11
**WGA**	636.04	372.98	223.66	592.63	494.12	987.44	626.13
**Marker Pair**	**UC1**	**UC2**	**UC3**	**UC4**	**UC5**	**UC6**	**UC7**
**ATF6**	706.87	411.92	421.99	599.38	277.71	434.69	1224.38
**BetaTubulin**	1250.36	838.92	809.9	1348.87	690.34	1107.8	2171.82
**ConcanavalinA**	789	526.44	588.51	1241.91	401.95	600	1221.41
**DAPI**	239.54	218.48	224.92	217.11	202.73	236.91	278.46
**GOLPH4**	725.69	499.68	590.89	220.42	269.98	456.84	485.73
**HSP60**	1671.15	854.78	775.68	1174.6	511.22	982.07	1895.63
**Nucleolin**	167.38	154.58	96.8	85.34	86.67	25.81	178.18
**Phalloidin**	949.26	451.44	498.97	1068.1	308.68	953.85	1273.32
**Sortilin**	990.55	505.19	397.59	1167.73	423.1	577.23	1374.41
**TOM20**	858.92	482.38	649.79	879.06	393.58	583.23	1130.1
**WGA**	870.18	483.5	483.86	767.4	318.4	564.18	1079.46

Supplementary Table 11. Major axis length (in pixels) of selected markers for 7 BM and UC MSCs obtained from CellProfiler (corresponding to Figure 4C).

Cell	TOM20 vs HSP60		GOLPH4 vs Sortilin	
	BM	UC	BM	UC
1	4.9E-106	0	8.1E-193	0
2	0	0	2.5E-230	0
3	0	8.9E-288	0	0
4	3.61E-30	1.08E-21	0	0
5	1.05E-67	0	0	0
6	1.3E-190	0	0	0
7	4.41E-91	0	0	0

Supplementary Table 12. Raw p-values obtained from the KS Hypothesis test for TOM20 vs HSP60 and GOLPH4 vs Sortilin (corresponding to Figure 4D).

References

1. Cappello, F., Conway de Macario, E., Marasà, L., Zummo, G. & Macario, A. J. L. Hsp60 expression, new locations, functions, and perspectives for cancer diagnosis and therapy. *Cancer Biology & Therapy* **7**, 801–809 (2008).
2. Merendino, A. M. *et al.* Hsp60 Is Actively Secreted by Human Tumor Cells. *PLOS ONE* **5**, e9247 (2010).
3. Recombinant Anti-Hsp60 antibody [EPR18245-93] - BSA and Azide free (ab224528). https://www.abcam.com/Hsp60-antibody-EPR18245-93-BSA-and-Azide-free-ab224528.html.
4. Bhagawati, M. *et al.* The receptor subunit Tom20 is dynamically associated with the TOM complex in mitochondria of human cells. *MBoC* **32**, br1 (2021).
5. Anti-Tom20 Antibody (F-10). https://www.scbt.com/p/tom20-antibody-f-10.
6. Hillary, R. F. & FitzGerald, U. A lifetime of stress: ATF6 in development and homeostasis. *Journal of Biomedical Science* **25**, 48 (2018).
7. Recombinant Anti-ATF6 antibody [EPR22690-84] - BSA and Azide free KO Tested (ab263955). https://www.abcam.com/atf6-antibody-epr22690-84-bsa-and-azide-free-ab263955.html.
8. Lujan, P. & Campelo, F. Should I stay or should I go? Golgi membrane spatial organization for protein sorting and retention. *Archives of Biochemistry and Biophysics* **707**, 108921 (2021).
9. Recombinant Anti-GOLPH4/GPP130 antibody [EPR13439] KO Tested (ab197595) | Abcam. https://www.abcam.com/golph4gpp130-antibody-epr13439-ab197595.html.
10. Kjolby, M., Nielsen, M. S. & Petersen, C. M. Sortilin, Encoded by the Cardiovascular Risk Gene SORT1, and Its Suggested Functions in Cardiovascular Disease. *Curr Atheroscler Rep* **17**, 18 (2015).
11. Recombinant Anti-Sortilin/NT3 antibody [EPR23093-58] - BSA and Azide free (ab263873). https://www.abcam.com/sortilinnt3-antibody-epr23093-58-bsa-and-azide-free-ab263873.html.
12. Concanavalin A, Alexa Fluor 488 Conjugate. https://www.thermofisher.com/order/catalog/product/C11252.
13. Bray, M.-A. *et al.* Cell Painting, a high-content image-based assay for morphological profiling using multiplexed fluorescent dyes. *Nat Protoc* **11**, 1757–1774 (2016).
14. Alexa Fluor 555 Phalloidin. https://www.thermofisher.com/order/catalog/product/A34055.
15. Wheat Germ Agglutinin, Alexa Fluor 647 Conjugate. https://www.thermofisher.com/order/catalog/product/W32466.
16. Recombinant Anti-Nucleolin antibody [EPR7952] - BSA and Azide free (ab226113). https://www.abcam.com/nucleolin-antibody-epr7952-bsa-and-azide-free-ab226113.html.
17. Anti-β Tubulin Antibody (D-10). https://www.scbt.com/p/beta-tubulin-antibody-d-10;jsessionid=g0cw-4HJEYyJsQi4LQp75hxE-9PtyQdjUrVTyytURt6f_laA6hUh!1267813408.
